# Rational Molecular Design of Redox‐Active Carbonyl‐Bridged Heterotriangulenes for High‐Performance Lithium‐Ion Batteries

**DOI:** 10.1002/advs.202306680

**Published:** 2023-12-03

**Authors:** Xipeng Shu, Liang Hu, Thomas Heine, Yu Jing

**Affiliations:** ^1^ Jiangsu Co‐Innovation Centre of Efficient Processing and Utilization of Forest Resources College of Chemical Engineering Nanjing Forestry University Nanjing 210037 China; ^2^ TU Dresden Fakultät für Chemie und Lebensmittelchemie Bergstraße 66c 01062 Dresden Germany; ^3^ Helmholtz‐Zentrum Dresden‐Rossendorf Forschungsstelle Leipzig Permoserstraße 15 04318 Leipzig Germany; ^4^ Department of Chemistry Yonsei University and ibs‐cnm Seodaemun‐gu Seoul 120‐749 Republic of Korea

**Keywords:** carbonyl‐bridged heterotriangulenes, cathode materials, first‐principles calculations, high redox‐potential, lithium‐ion batteries

## Abstract

Carbonyl aromatic compounds are promising cathode candidates for lithium‐ion batteries (LIBs) because of their low weight and absence of cobalt and other metals, but they face constraints of limited redox‐potential and low stability compared to traditional inorganic cathode materials. Herein, by means of first‐principles calculations, a significant improvement of the electrochemical performance for carbonyl‐bridged heterotriangulenes (CBHTs) is reported by introducing pyridinic N in their skeletons. Different center atoms (B, N, and P) and different types of functionalization with nitrogen effectively regulate the redox activity, conductivity, and solubility of CBHTs by influencing their electron affinity, energy levels of frontier orbitals and molecular polarity. By incorporating pyridinic N adjacent to the carbonyl groups, the electrochemical performance of N‐functionalized CBHTs is significantly improved. Foremost, the estimated energy density reaches 1524 Wh kg^−1^ for carbonyl‐bridged tri (3,5‐pyrimidyl) borane, 50% higher than in the inorganic reference material LiCoO_2_, rendering N‐functionalized CBHTs promising organic cathode materials for LIBs. The investigation reveals the underlying structure‐performance relationship of conjugated carbonyl compounds and sheds new lights for the rational design of redox‐active organic molecules for high‐performance lithium ion batteries (LIBs).

## Graphical abstract

Incorporation of pyridinic N by forming pyrimidine rings in the skeletons of carbonyl‐bridged heterotriangulenes (CBHTs), is a rational strategy to obtain organic functional materials with high redox activity, high theoretical capacity, suppressed solubility and enhanced charge transport ability. The designed N‐functionalized CBHTs are promising candidates to replace traditional transition‐metal‐containing inorganic cathodes for high‐performance lithium‐ion batteries.

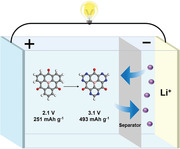

## 1 Introduction

The utilization of renewable energy sources including solar and wind energy to generate electricity is an imperative means to alleviate the energy crisis and environmental pollution caused by excessive burning of fossil fuels,^[^ ^1^, ^2^, ^3^, ^4^ ^]^ but it requires the application of scalable energy storage systems. Secondary batteries represented by lithium‐ion batteries (LIBs) can realize scalable storage of intermittent renewable energy and are the most widely used power supply devices for commercial electronics and electric vehicles.^[^ ^5^ ^]^ However, the high dependence of traditional inorganic electrode materials on compounds (e.g., LiCoO_2_, LiMn_2_O_4_) containing expensive and toxic transition metals (e.g., Co, V, Mn) is a major problem, challenging the sustainable development of LIBs.^[^ ^6^, ^7^ ^]^ Especially, the requirement of large amounts of cobalt for batteries has led to serious environmental and social issues, including child labor. Therefore, developing metal‐free electrode materials can contribute to a societally and environmentally benign scale‐up of battery production.

Organic materials have genuine advantages over their inorganic counterparts as sustainable electrode candidates.^[^ ^8^ ^]^ Owing to their structural diversity they are tunable for specific functionality, they do not include metals or other precious or toxic elements, they are of low weight, and potentially cost‐effective.^[^ ^9^, ^10^, ^11^, ^12^ ^]^ In the past decades, great efforts have been devoted to developing redox‐active organic materials for LIBs.^[^ ^13^ ^]^ Among them, organic carbonyl compounds have attracted extensive research interests because of their rapid kinetics and high reversibility of redox reactions with Li ions during charge/discharge cycles.^[^ ^14^ ^]^ For instance, quinone derivatives, which are highly abundant in natural plants, have been widely studied as electrode materials for LIBs.^[^ ^15^ ^]^ Because of its low molecular mass, 1,4‐benzoquinone shows a high theoretical capacity of 496 mAh g^−1^ at the discharge voltage of 2.8 V (vs Li^+^/Li).^[^ ^16^ ^]^ However, the high solubility in organic electrolytes and low conductivity of carbonyl compounds result in severe capacity decay.^[^ ^17^ ^]^ In addition, the relatively low redox potential of carbonyl organic electrodes compared with inorganic materials has been a bottleneck for their use as LIB cathodes.^[^ ^18^, ^19^ ^]^ Therefore, alternative organic cathode materials with high redox potential and high specific capacity are highly demanded. Meanwhile, effective design strategies to comprehensively reduce the solubility and enhance the redox‐activity, capacity and conductivity of carbonyl compounds are of great significance for realizing sustainable high‐performance LIBs.^[^ ^20^, ^21^, ^22^ ^]^

Previous experimental studies have demonstrated that a highly conjugated π system is beneficial to improve the electrical conductivity of carbonyl compounds. At the same time, it inhibits their dissolution in organic electrolyte, thereby enhancing the rate capability and long‐term cyclability of LIBs.^[^ ^23^, ^24^ ^]^ For example, Yang et al. demonstrated that the large near‐planar scaffolds of 1,4‐bis(p‐benzoquinonyl) benzene and 1,3,5‐tris(p‐benzoquinonyl) benzene lower the solubility in electrolyte of 1,3‐dioxolane (DOL) and 1,2‐dimethoxyethane (DME) (1:1 by volume) compared to p‐benzoquinone.^[^ ^25^ ^]^ Yoshida et al. revealed that higher aromaticity of pyrene‐4,5‐dione resulted in better rate performance and structural stabilization compared to acenaphthenequinone and benzocyclobutenedione.^[^ ^26^ ^]^ These examples demonstrate that high‐performance organic electrodes can be discovered by exploring organic molecules with large aromatic skeletons and abundant redox‐active sites.

**Scheme 1:**
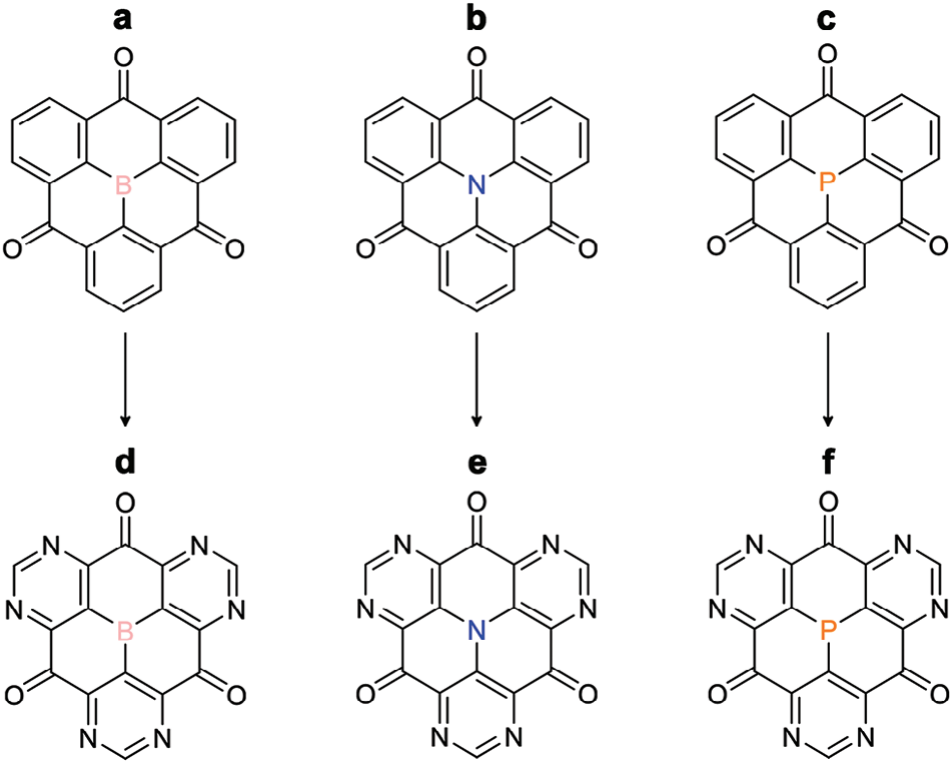
Incorporation of pyridinic‐N in the aromatic skeleton of a) carbonyl‐bridged triphenylborane (CTPB), b) carbonyl‐bridged triphenylamine (CTPA) and c) carbonyl‐bridged triphenylphosphine (CTPP) to form d) carbonyl‐bridged tri (3,5‐pyrimidyl) borane (CTPB(3,5‐pyrimidyl)), e) carbonyl‐bridged tri (3,5‐pyrimidyl) amine (CTPA(3,5‐pyrimidyl)) and f) carbonyl‐bridged tri (3,5‐pyrimidyl) phosphine (CTPP(3,5‐pyrimidyl)), respectively.

Recently, heterotriangulenes, which were originally proposed by Hellwinkel et al., have attracted wide attentions because of their strong electron conjugation and tunable molecular structures.^[^ ^27^, ^28^ ^]^ Different heteroatom‐centered (including B, N, P) heterotriangulenes have been synthesized and show potential applications in organic light‐emitting diodes and fluorescence emitters.^[^ ^29^, ^30^ ^]^ Their D_3h_ symmetry renders them as ideal building monomers of 2D conjugated polymers for photocatalysis, which can be optimized by taking advantage of their rich chemical variety.^[^ ^31^, ^32^, ^33^ ^]^ A particularly interesting group of heterotriangulenes are those with bridging carbonyls (**Scheme** **1**a–c), which are readily obtained using the Friedel–Crafts approach. These carbonyl‐bridged heterotriangulenes (CBHTs) exhibit three redox‐active carbonyl groups and highly conjugated π‐orbitals,^[^ ^34^, ^35^ ^]^ and thus are expected to be attractive organic cathode materials for LIBs. Moreover, multiple structure modifications are possible to further tune the properties of these molecules toward better performance.^[^ ^36^ ^]^ Despite these interesting and motivating properties, no research has reported the use of CBHTs as cathode materials, and the relationship between the redox activity and composition of these molecules remains an open question.

Herein, by using first‐principles calculations, we have explored the potential use of CBHTs as organic cathode materials for LIBs. As candidate structures, we included CTPB, CTPA, and CTPP (Scheme 1a–c). Examination of the structural and electronic properties and redox activities of CBHTs indicate that different center atoms significantly influence the Li binding capability of the molecules. We propose the incorporation of pyridinic‐N to the aromatic skeletons as an effective strategy to improve the redox activity of the molecules by increasing their electron affinity (Scheme 1d–f). In this way, the redox potentials are significantly increased and the theoretical capacities of CBHTs double compared to those of the original unfunctionalized moieties. The structure‐performance relationship of different N‐functionalized CBHTs and the effects of solvation and dissolution as important factors in influencing the redox potential of CBHTs were explored. We found that the electrochemical performance of CBHTs can be comprehensively improved by the incorporation of pyridinic N, as it effectively increases the electron deficiency of the central region of the molecule and enhances the molecular polarity. With high theoretical capacity of 493, 489, and 468 mAh g^−1^, high redox potential of 3.09, 2.56, and 2.81 V, enhanced conductivity and suppressed solubility, CTPB/A/P(3,5‐pyrimidyl) (Scheme 1d–f) are proposed as promising organic cathode materials for high‐performance LIBs with high energy density of 1524, 1251, and 1306 Wh kg^−1^, respectively, surpassing that of traditional cathode materials, that is, LiMn_2_O_4_ (500 Wh kg^−1^), LiFePO_4_ (587 Wh kg^−1^) and LiCoO_2_ (1000 Wh kg^−1^).^[^ ^37^ ^]^

## 2 Results and Discussion

### 2.1 Structure, Electronic Properties, and Redox Activity of CBHTs

**Figure 1:**
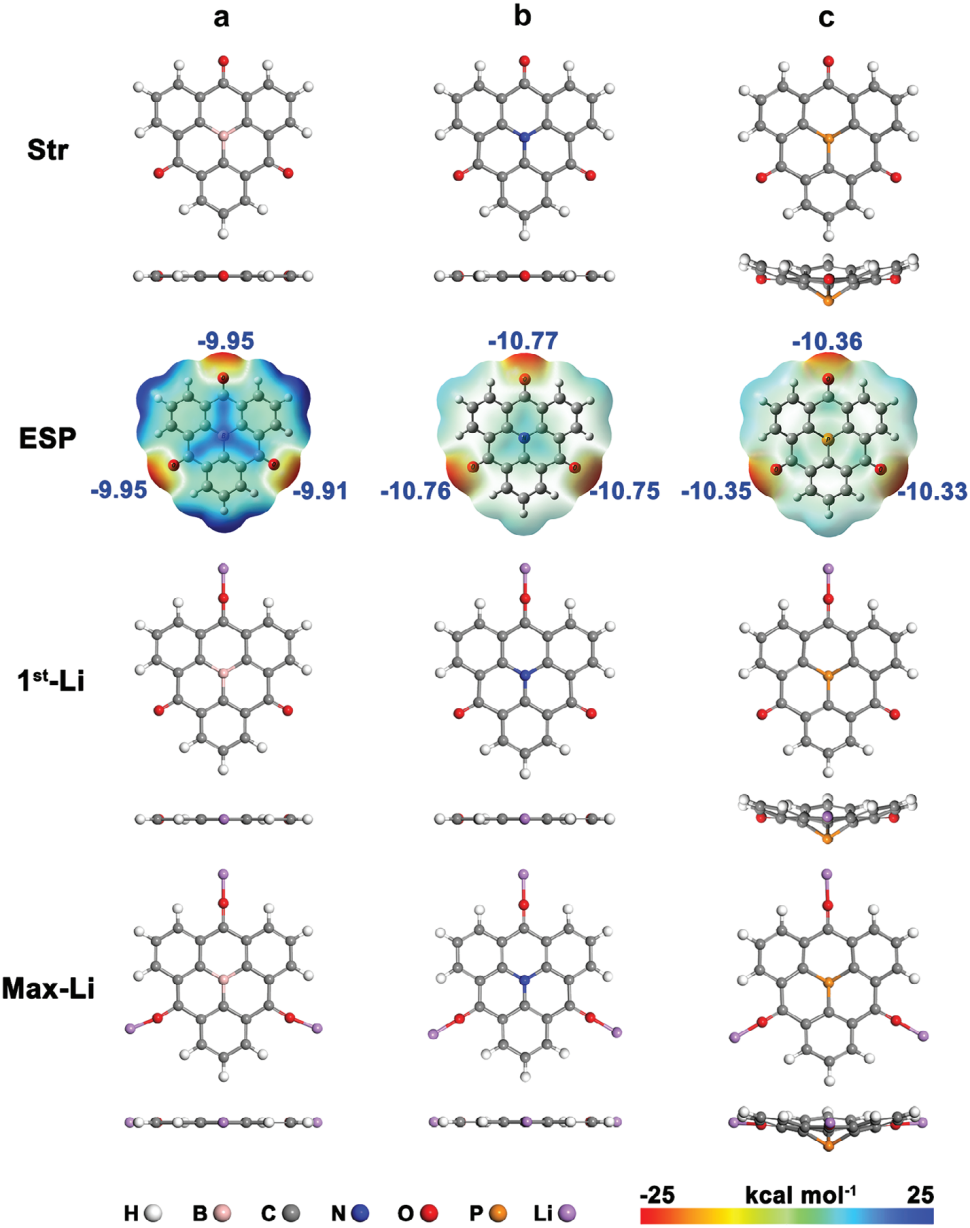
Optimized molecular structures, MESP distribution and structures combined with one Li (1st‐Li) at the optimal site and with the maximum amount of Li (Max‐Li) for a) CTPB, b) CTPA, c) CTPP. The MESP values follow the scale −25–25 kcal mol^−1^ at color bar, red areas are electron‐rich, while blue regions are electron‐deficient.

The optimized structures of CTPB/A/P are shown in **Figure** **1**. Note that the synthesis of N‐center possessing CTPA has been reported,^[^ ^38^ ^]^ while for B and P centered HTs the derivatives with other bridging groups (e.g., O and S instead of carbonyls) are known experimentally.^[^ ^39^, ^40^ ^]^ Thus, the synthesis of CTPB/P could follow established synthesis strategies (e.g., through the Friedel–Crafts approach).^[^ ^34^ ^]^ Because of the high π‐electron delocalization within the carbon skeletons, CTPB/A show planar configurations. In contrast, as the central C─P bond length (1.80 Å) is much larger than those of C─B (1.52 Å) and C─N (1.41 Å), CTPP has a bowl‐shaped structure. The three carbonyl (C═O) bridging functional groups in each CBHT molecule act as active sites to accommodate lithium. The highest occupied molecular orbital (HOMO) and lowest unoccupied molecular orbital (LUMO) energy level and HOMO‐LUMO gap are presented in Table S1 and Figure S2 (Supporting Information). The CBHTs strongly differ in their electronic properties, as the central heteroatoms differently impact the degree of electron delocalization and change the shape of the frontier orbitals. The LUMO energy level of CTPB is lower than that of CTPA and CTPP, and the energy gap of CTPB (3.63 eV) is smaller than that of CTPA (3.80 eV) and CTPP (4.19 eV).

We have identified the preferred Li binding sites by analyzing the molecular electrostatic potential (MESP) distribution in the molecules.^[^ ^41^, ^42^, ^43^ ^]^ Generally, the higher the MESP, the more active the site is. For the CBHTs, the MESP has a minimum at the three carbonyl groups, given by the electron‐rich lone pairs of the O atoms, which are the most preferred binding sites for electrophilic Li (Figure 1). We also note the characteristic MESP distribution around the center atom, with electron deficient B center because of its empty p_z_, and electron‐rich N and P centers with filled p_z_ orbital. The 1st Li binding energy at the CBHT carbonyl redox sites (details are given in Equation S1 and Table S2, Supporting Information) accounts to −1.78, −1.43 and −1.40 eV for CTPB/A/P, respectively.

The binding energy per Li decreases with increasing number of binding Li atoms (Table S3, Supporting Information). As a result, the redox potential for the 1st–3rd carbonyl gradually decreases (Equations S2 and S3 and Figure S3, Supporting Information). Since previous experimental study indicated that oxygen‐containing functional groups can react rapidly and reversibly with Li through a synchronized mechanism^[^ ^44^ ^]^ and the three carbonyl groups of CBHTs have identical chemical environment, the target molecules will combine with Li through a synchronization mechanism. The average redox potential and theoretical specific capacity were calculated by assuming that all three carbonyl groups are equivalently combined with Li (Equation S4 and Table S2, Supporting Information). The average redox potential was revealed to be 2.08, 1.83, and 1.79 V (vs Li^+^/Li) for CTPB/A/P respectively, showing a significant impact of the center atoms on the redox activity of the carbonyl groups. Although, the theoretical capacity of CBHTs (236–251 mAh g^−1^) is comparable to many known carbonyl compounds (Table S4, Supporting Information), the lower redox potentials will limit their application as LIB cathodes. Thus, effective strategies are needed to further enhance their redox activity to make them competitive cathode materials with high redox potential for LIBs.

### 2.2 Role of Pyridinic‐N in Improving the Redox Activity of CBHTs

Previous studies have demonstrated that the introduction of electron‐deficient substituents to the quinone skeletons can be a feasible strategy to tune their redox activity.^[^ ^45^, ^46^, ^47^ ^]^ However, the increased molecular mass caused by the added functional groups contributes to decreased mass specific capacities. In contrast, introducing heteroatoms into the skeletons can be an efficient method to enhance the redox activity of organic molecules without severely changing their molecular mass.^[^ ^48^, ^49^, ^50^ ^]^ In light of the fact that π‐conjugated N‐containing heteroaromatic molecules have high redox activity to combine Li, introducing highly nucleophilic pyridinic‐N^[^ ^24^ ^]^ into the aromatic rings of CBHTs can be a feasible strategy to further improve their redox activity. This way, the high aromaticity of the molecules can be maintained while the molecular mass is hardly changed. Herein, we constructed N‐functionalized CBHTs with pyridine and pyrimidine units and explored their structural and electronic properties and electrochemical performance for Li storage.

**Figure 2:**
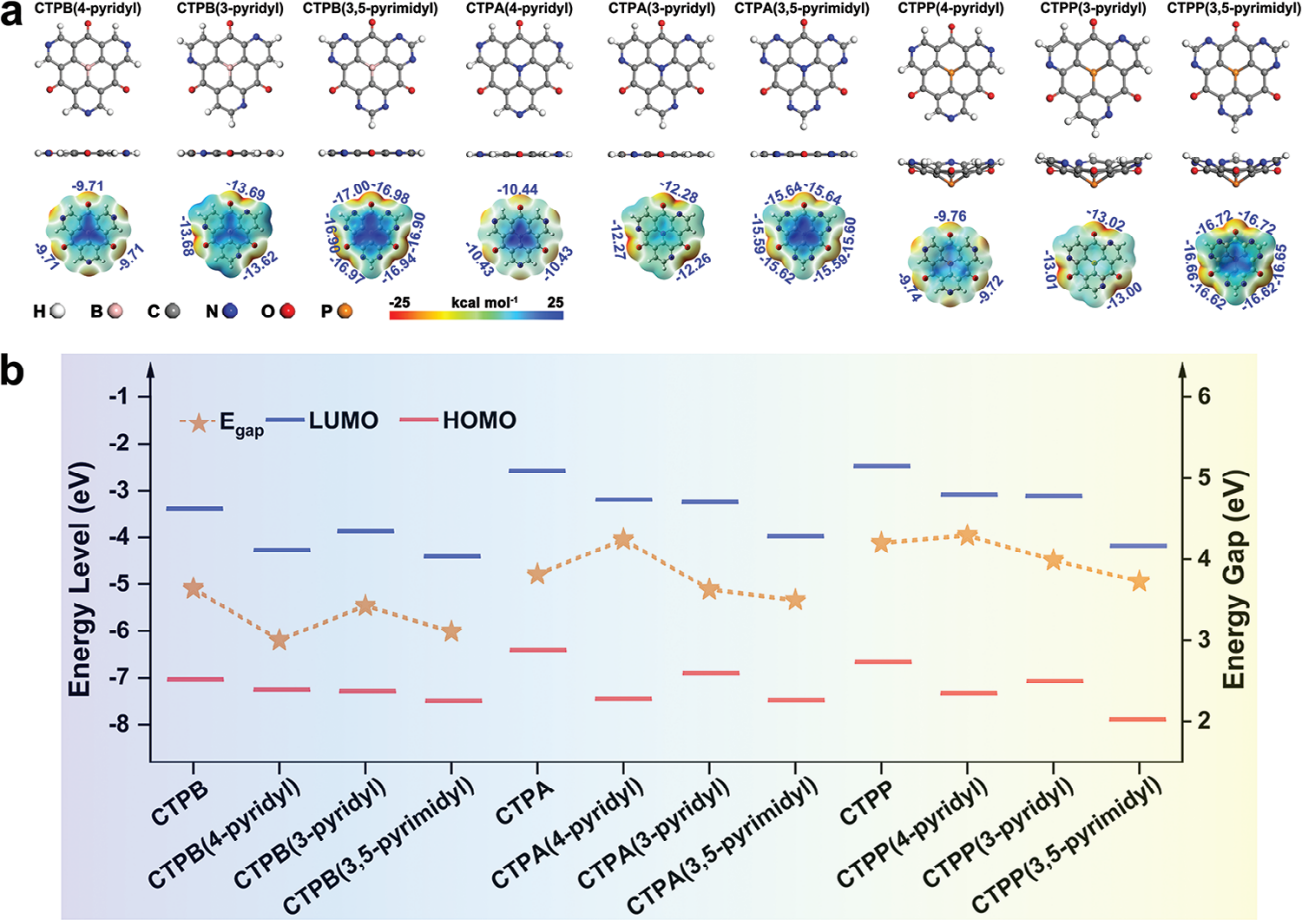
a) Optimized molecular structures and MESP distribution of N‐functionalized CBHTs. The MESP values follow the scale −25–25 kcal mol^−1^ at color bar, and the red color and blue color represent a negative and positive electronic potential, respectively. b) The frontier orbital energy levels (eV) and HOMO‐LUMO gaps (eV) of CBHTs and their N‐functionalized derivatives.

As shown in **Figure** **2**a, there are two possible positions for introducing one pyridinic‐N, while there is one configuration for pyrimidine in CBHTs, where at least the C_3_ symmetry of the molecules is maintained. Geometry optimization shows very little structural impact (see Figure 2), however, as shown in Figure 2b, except for CTPA(4‐pyridyl) and CTPP(4‐pyridyl), the HOMO‐LUMO gaps decrease by 0.18–0.64 eV (see Table S5, Supporting Information). This indicates enhanced electron conjugation and is beneficial for their application in LIBs. In addition, the position and number of incorporated N atoms affects the distribution of electrons and change the level of the frontier orbitals. Generally, N substitution at the meta‐position of carbonyls leads to a more significant downshift of the energy levels of the frontier orbitals than the substitution at the adjacent position, while increasing the content of N‐functionalization at the adjacent position results in a further decrease in the HOMO‐LUMO gap of CBHTs. The LUMO level shifts downward after incorporating N, which will further affect their redox properties for Li combination. As shown in Figure S4 (Supporting Information), the incorporation of pyridinic N destroys the electron density uniformity of benzene and the MESP minima move to the electron‐rich N sites. Therefore, the introduction of pyridinic‐N to the aromatic ring can be an efficient and general strategy to tune the electronic properties of aromatic molecules,^[^ ^51^, ^52^ ^]^ which will further affect their redox activity.

In the plotted MESP maps of the different pyridinic N‐functionalized CBHTs (Figure 2a) the incorporated N atoms determine the local MESP minima and are expected to be active sites to combine with Li. Regardless of the center atom, molecules with N adjacent to the carbonyl group show more negative MESP minima than those with N functionalized at the meta‐position, because of the additive effect of electronegative N to O. Interestingly, when the pyridinic‐N is located adjacent to the carbonyl group, the potential minima are located between N and O (Figure 2a), indicating that Li will bind simultaneously to both N and O. Furthermore, severe electrostatic potential differences are observed when the aromatic benzyl is replaced by the pyrimidyl group. The central area is more electron‐deficient, while the carbonyl group incorporated with two pyridinic N is more electron‐rich.

The Li binding energies (Table S6, Supporting Information) at the redox‐active sites are in range of −1.66–−3.30 eV for CTPB/A/P(4‐pyridyl), CTPB/A/P(3‐pyridyl) and CTPB/A/P(3,5‐pyrimidyl). The pyridinic‐N adjacent to the carbonyl group works synergistically with O to bind with Li (Figure S5, Supporting Information) and results in a stronger binding energy than the precursor molecules (Table S2, Supporting Information) and molecules with pyridinic‐N located at the meta‐position. Moreover, as shown in Figure S6 (Supporting Information), there is a linear relationship between the Li‐binding strength and the MESP value for each N‐functionalized CBHT.^[^ ^53^ ^]^ A deeper MESP minimum corresponds to a stronger binding energy at the examined site. Therefore, the MESP analysis indicates both the binding site and binding strength of Li. Note that the feasibility of Li accommodation at the MESP predicted binding sites have been confirmed by a further calculation of the Li binding energy (see details in Table S7 and Figure S9, Supporting Information). Remarkably, for CTPB/A/P(3,5‐pyrimidyl) that possess three pyrimidyl groups, there are six equivalent Li‐binding sites, twice as many as in the original molecules, which will double the theoretical Li storage capacity.

### 2.3 Electrochemical Performance of Pyridinic N‐Functionalized CBHTs for LIBs

First, we calculated the average redox potential (Table S6, Supporting Information) by assuming that all the active sites were occupied by Li (Figure S7, Supporting Information). It ranges from 1.84 to 3.09 V (vs Li^+^/Li) after fully discharging (Figure S8, Supporting Information). As expected, the redox potential is significantly increased after incorporating pyridinic N, and molecules with N adjacent to the carbonyls show the highest redox potential, almost 1.0 V higher than the original CBHTs. Remarkably, for molecules substituted with pyrimidyl groups, where the full discharge state is considered by combining even six Li atoms, the redox potential remains as high as 3.09, 2.56, and 2.81 V for CTPB/A/P(3,5‐pyrimidyl), respectively. This high redox activity stems from the enhanced nucleophilicity of the bridge groups and increased electron‐deficiency of the central region induced by pyridinic N.^[^ ^54^ ^]^ This result is also in good accordance with previous studies, which indicated that molecules with a larger area of electron deficient space in the central backbone have a higher redox potential.^[^ ^25^, ^26^, ^55^ ^]^ Moreover, CTPB (3,5‐pyrimidyl) exhibits a higher redox potential (3.09 V) than CTPA (3,5‐pyrimidyl) (2.56 V) and CTPP (3,5‐pyrimidyl) (2.81 V), which again is rationalized with the high electron‐deficiency of the empty p_z_ orbital of B. The highly enhanced redox potential of N‐functionalized CBHTs will make them competitive cathode candidates for LIBs.

**Figure 3:**
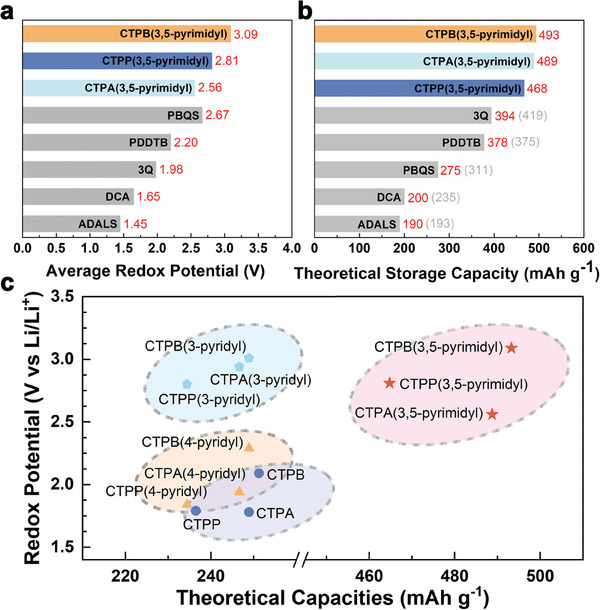
a) Average redox potential and b) Theoretical storage capacity of CTPB(3,5‐pyrimidyl) in comparison with that of other organic cathode materials. Note that we also have calculated the theoretical specific capacities (provided with gray numbers in the brackets) of those experimentally known molecules, which agree well with the experimental results (Table S9, Supporting Information). c) The theoretical capacities and redox potentials of CBHTs and their N‐functionalized derivatives.

The theoretical specific capacities range from 234 to 249 mAh g^−1^ for one pyridinic‐N functionalized molecules, and for the pyrimidyl‐substituted molecules even between 468 and 493 mAh g^−1^. The mass energy density is as high as 1524, 1251, and 1306 Wh kg^−1^, respectively, for CTPB/A/P(3,5‐pyrimidyl). Thus, these molecules outperform most of the experimentally reported organic cathode materials (**Figure** **3**a,b; Table S8, Supporting Information), such as poly (benzoquinonyl sulfide) (PBQS),^[^ ^56^ ^]^ poly[1,4‐di(1,3‐dithiolan‐2‐yl)benzene)] (PDDTB),^[^ ^57^ ^]^ fused N‐heteroaromatic triquinoxalinylene molecules (3Q),^[^ ^24^ ^]^ 9,10‐dicyanoanthracene (DCA)^[^ ^58^ ^]^ and Azobenzene‐4,4′‐dicarboxylic acid lithium salt (ADALS).^[^ ^59^ ^]^

### 2.4 Solvation Effect and Solubility of CBHTs and their N‐Functionalized Derivatives

The redox potential of CBHTs and their N‐functionalized derivatives were calculated by considering the solvation effect of the electrolyte (EC and DMC, 3:7 by volume, *ε*_r_ = 16.14). To better understand the influence of solvation effect, we compared the redox potentials of the title compounds with those calculated at the vacuum state. The calculated redox potential in vacuum is smaller than that in solvent, which agrees with the findings in our previous work.^[^ ^60^ ^]^ This can be understood because organic solvents containing EC and DMC show strong dielectric polarization^[^ ^61^ ^]^ and the strong intermolecular interactions in the dielectrically polarized environment will help stabilize the redox‐active molecules after lithiation.^[^ ^62^ ^]^ We found that after N‐functionalization the redox potential of CBHTs is less influenced by the electrolyte that that of the precursor molecules (CTPB/A/P) (Figure S11, Supporting Information). For example, for CTPB/A/P (3,5‐pyrimidyl), the redox potential calculated at vacuum and in solutions of DM, EC/DMC and TMU, is nearly the same, Therefore, we can conclude that indicating that N‐functionalization can lead to robust redox activity and high stability of CBHTs in the electrolyte (Table S10, Supporting Information). Therefore, these molecules are expected to work efficiently in most common solvent environments as cathodes for LIBs.

**Figure 4:**
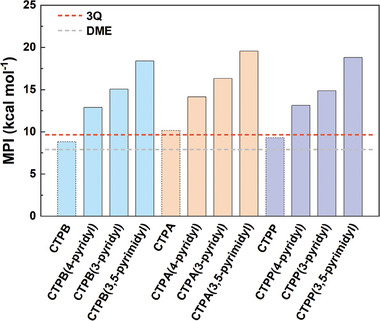
The molecular polarity index (MPI, kcal mol^−1^, Equation S5, Supporting Information) values of CBHTs and their N‐functionalized derivatives. The red and orange dashed lines indicate the MPI values of 3Q (9.64 kcal mol^−1^) and DME (7.92 kcal mol^−1^), respectively. Note that the 3Q molecule exhibits good stability in DME solution.^[^ ^24^ ^]^

Next, we estimated the solubility of CBHTs and their derivatives in the non‐protonic electrolyte because dissolution of organic materials in electrolytes is a main problem that causes capacity degradation. The fused N‐heteroaromatic triquinoxalinylene molecule has been demonstrated to show good stability in ether‐based electrolytes (e.g., DME) because of the large conjugated structure,^[^ ^24^ ^]^ which exhibits a similar skeleton to those of our studied molecules. According to the similarity‐solubility (“like dissolves like”) principle, solubility of different organic molecules in the electrolyte can be evaluated by estimating their polarity differences. We estimated the polarity of CBHTs and their derivatives by using the value of MPI as an indicator.^[^ ^63^ ^]^ As shown in Table S11 (Supporting Information), the MPI values of CBHTs and their N‐functionalized derivatives range from 8.81 to 19.55 kcal mol^−1^, while the MPI value for the referenced solvent (DME) was 7.92 kcal mol^−1^. Compared with original CBHTs, the polarities of their pyridinic‐N functionalized derivatives are more different from those of DME (**Figure** **4**). Therefore, it can be expected that the N‐functionalized CBHTs should have lower solubility in the electrolyte than the original molecules, leading to better cycling stability when used as cathodes for LIBs.

As mentioned at the beginning, our previous investigations have demonstrated the feasibility of HTs to form 2D polymers.^[^ ^31^, ^32^, ^33^ ^]^ Thus, we further explored the potential of 2D polymers made of CTPB(3,5‐pyrimidyl) as electrode materials for LIBs (Figure S12a, Supporting Information) because 2D CTPB(3,5‐pyrimidyl) with extended π‐conjugation will definitely show enhanced conductivity and reduced solubility in liquid electrolyte. We found that the carbonyl groups are also the redox active site to bind with Li, which can accommodate up to 24 Li atoms (Figure S12b, Supporting Information) for 2D CTPB (3,5‐pyrimidyl) with a high theoretical capacity of 1065 mAh g^−1^. However, the average open circuit voltage is 0.87 eV, indicating that 2D CTPB(3,5‐pyrimidyl) can be used as a promising non‐metal anode material for LIBs. Since liquid electrolytes are flammable and can trigger the formation of lithium dendrite during cycling, using solid electrolytes, such as Li_1.5_Al_0.5_Ge_1.5_(PO_4_)_3_ (LAGP) electrolyte,^[^ ^64^ ^]^ can be an alternative solution to improve the electrochemical performance of organic electrode materials.

### 2.5 Charge Transport Ability of CBHTs and their N‐Functionalized Derivatives

Since the low solubility of CBHTs in the electrolyte helps to maintain their high crystallinity when used as cathodes, the conductivity of these molecules is a further important aspect for evaluating their performance for LIBs. As discussed above, the HOMO‐LUMO gap of CBHTs decreases after incorporating pyridinic N in the aromatic rings, indicating that their N‐functionalized derivatives enhance electron conjugation. Moreover, as shown in Figure S13 (Supporting Information), the HOMO‐LUMO gap of all examined molecules is significantly reduced after lithiation, indicating that the combination of Li on the redox active sites of CBHTs and their derivatives will further facilitate electron delocalization along the molecules. Finally, we examined the carrier mobility (μ) of these molecules to better evaluate their charge transport performance for LIBs (Equations S6–S10, Supporting Information). Generally, the reorganization energy (λ) is one of the key factors to determine the efficiency of charge transport in the hopping transport system.^[^ ^65^ ^]^ Organic semiconductors with low λ usually exhibit a high charge mobility.^[^ ^66^ ^]^ The λ for the target molecules are calculated and listed in **Table** **1**, in comparison with the well‐known organic semiconductor, pentacene (PEN).^[^ ^67^ ^]^ CTPB(3,5‐pyrimidyl) shows a λ value of 116 meV, which is even smaller than that of PEN (130 meV), indicating a good charge transport ability.

**Table 1:** The calculated reorganization energy (λ), transfer integral (V) and carrier mobility (μ) for CTPB(3,5‐pyrimidyl), CTPB and CTPA, respectively.

Molecules^a)^	λ [meV]	V [meV]	μ [cm^2^V^−1^s^−1^]	References
CTPB(3,5‐pyrimidyl)	116	327	5.27	This work
CTPB	138	194	1.62	This work
CTPA	164	92	0.33	This work
PEN	130	‐	‐	[67]
PFP	‐	‐	0.47	[68]

^a)^ Note that to validate the reliability of the employed computational methods, we have calculated the reorganization energy of PEN, which turns out to be 131 meV, in good agreement with a previous theoretical study using similar method.^[^ ^67^ ^]^ This result demonstrates the reliability of our prediction for the charge transport properties of CBHTs. The details about the calculation of reorganization energy (λ), transfer integral (V) and carrier mobility (µ) are given in the Supporting Information.

For organic compounds, the charge transport process is accomplished through intermolecular interactions between the neighboring molecules. We examined the charge transport performance of CTPA that has been experimentally synthesized.^[^ ^38^ ^]^ The crystal structure shows that the molecules are arranged in a classic herringbone pattern with the columnar π‐stacking mode. The various types of charge hopping pathways for CTPA are shown in Figure S14 (Supporting Information), including edge‐to‐face motif, π‐stacking motif and edge‐to‐edge motif (Figure S15, Supporting Information). The results show that the π‐stacking motif has the highest transfer integral (V). The other pathways exhibit weaker intermolecular interactions because of the large inter‐molecule distance. μ is calculated based on the values of λ and V obtained above (Equation S8, Supporting Information), which is 0.33 cm^2^V^−1^s^−1^ for CTPA, comparable to that (0.47 cm^2^V^−1^s^−1^) of perfluoropentacene (PFP) as reported before.^[^ ^68^ ^]^

Since CTPB and CTPB(3,5‐pyrimidyl) have not been experimentally synthesized, the molecular stacking pattern of these two molecules were predicted using DFT (Figure S14, Supporting Information). By showing a planar molecular structure, both CTPB and CTPB(3,5‐pyrimidyl) form a parallel offset π‐stacking structure. Notably, the π‐stacking rods are piled up on each other with a very tight packing structure, which is favorable for fast charge transport.^[^ ^39^ ^]^ The charge hopping pathways for CTPB and CTPB(3,5‐pyrimidyl) are illustrated in Figures S16 and S17 (Supporting Information). As exhibited in Table 1, the carrier mobility (5.27 cm^2^V^−1^s^−1^) of CTPB(3,5‐pyrimidyl) is much higher than that of CTPA and CTPB, as the functionalization of pyridine N enhances the intermolecular interactions of CBHTs, thus reducing the reorganization energy, increasing the transfer integral and enhancing the carrier mobility. This finding is in good accordance with a previous study.^[^ ^69^ ^]^ Therefore, it can be expected that CTPB and CTPB(3,5‐pyrimidyl) are appealing organic semiconductors for electronics, if they could be experimentally synthesized. Definitely, the enhanced charge transport ability of CTPB(3,5‐pyrimidyl) than CTPB is beneficial for its use in LIBs.

The examined molecules show good structural stability as their configurations are hardly affected after lithiation. As illustrated in Table S12 (Supporting Information), the central C‐B (C‐N or C‐P) bond and the bridging carbonyl (C═O) bond of CBHTs and their N‐functionalized derivatives change by <4.2 and 18.8 pm after lithiation, respectively. This can be ascribed to the high symmetry of the designed molecules, which will be beneficial to the flexible and reversible combination of Li at the carbonyl groups and the pyridinic‐N sites during charge/discharge process.

Note that the crystallization of organic molecules will inevitably influence the kinetic of redox reaction and therefore the performance of the assembled LIBs. However, MD simulations on interpreting the redox kinetics will be challenging due to the lack of reliable force field. We will focus on the kinetics of lithium ion diffusion in experimentally known organic crystals in our future studies.

## 3 Conclusion

We have explored the potential of using CBHTs as cathode materials for LIBs and found that different center atoms affect the redox activity of carbonyl groups and the high electron deficiency of B center makes CTPB more active to combine with Li than CTPA/P. Incorporation of pyridinic‐N in the aromatic rings significantly affects the redox activity of the molecule without significantly increasing the molecular mass, while the redox potential of CBHTs is significantly improved when pyridinic‐N is incorporated at the 3, 5 sites by forming pyrimidine rings. The improved redox activity is ascribed to the downshift of the LUMO energy level and increased electron deficiency of the central area. In addition, the lone pair of electrons in the sp^2^ orbital of the adjacent pyridinic‐N atom strengthens the coordination of Li at the carbonyl sites, which not only contribute to increased redox potentials but also double the theoretical capacities compared to the original CTPBs. As a result, the N‐functionalized CBHTs with pyrimidyl groups show high redox potentials and high theoretical capacities, and the energy density is predicted to be as high as 1524, 1251 and 1306 Wh kg^−1^ for CTPB/A/P(3,5‐pyrimidyl), respectively. The designed molecules show robust redox activity to the electrolyte, their solubility is highly suppressed and the charge transport ability is enhanced after N‐functionalization. With high stability, suppressed solubility and high energy density, the designed N‐functionalized CBHTs are predicted to be promising cathode materials for LIBs. Our investigation reveals the underlying correlation between the composition and electrochemical properties of carbonyl compounds and provides useful insight into the rational design of organic cathode materials for next generation LIBs. Regarding to the potential synthesis of N‐functionalized CBHTs, we propose a possible synthetic route for CTPB (3,5‐pyrimidinyl) (Scheme S1, Supporting Information). The key synthetic transformation for the N‐functionalized CBHTs relied on the acid‐promoted threefold intramolecular Friedel‐Crafts acylation reaction of triester. The reaction will be challenged with limited yield due to the competition of twofold carbonyl bridged side product generated by partial decarboxylation, which requires the efforts of organic chemists.

## 4 Experimental Section

### Computational Methods

Density functional theory (DFT) calculations were performed for CBHTs and their derivatives by using the Gaussian 16 package^[^ ^70^ ^]^ and Multiwfn.^[^ ^71^ ^]^ The structures were optimized by using the B3LYP‐D3 function with the standard 6–31G (d,p) basis set.^[^ ^72^ ^]^ The local minima were confirmed by computing the vibrational frequencies of the molecules at the same level. B3LYP‐D3 with the basis set of 6–311+G(d,p) was used to depict the total energy and electronic properties of the examined structures.^[^ ^73^ ^]^ The solvation free energies were evaluated by adopting the universal solvation model, i.e., the solvation model based on density (SMD).^[^ ^74^ ^]^ The mixture of ethylene carbonate (EC) and dimethyl carbonate (DMC) (3:7 by volume) with a dielectric constant of 16.14 was used as the electrolyte to estimate the solvation effect, which were commonly used as electrolyte solvents in LIBs.^[^ ^18^, ^45^ ^]^ By this way, the electrochemical performance of CBHTs could be visualized by directly comparing the redox potential and theoretical capacity with that reported in literature.

## Conflict of Interest

The authors declare no conflict of interest.

## Supporting information

Supporting Information

## Data Availability

The data that support the findings of this study are openly available in [NOMAD] at [https://nomad‐lab.eu/prod/v1/gui/user/uploads/upload/id/NyD6D‐sHQ1OttsS3‐Zfv3g], reference number 1.
