# Supplementary material for: Rational Molecular Design of Redox‐Active Carbonyl‐Bridged Heterotriangulenes for High‐Performance Lithium‐Ion Batteries
Source: Adv Sci (Weinh). 2023 Dec 3;11(6):2306680. doi: 10.1002/advs.202306680 (PMC10853723; doi:10.1002/advs.202306680)
Supplement: Supplementary file 1 — Supporting Information [file ADVS-11-2306680-s001.pdf]

ADVANCED SCIENCE
Open Access

Supporting Information

for *Adv. Sci.*, DOI 10.1002/advs.202306680

Rational Molecular Design of Redox-Active Carbonyl-Bridged Heterotriangulenes for High-Performance Lithium-Ion Batteries

Xipeng Shu, Liang Hu, Thomas Heine and Yu Jing**

Supporting Information

Rational Molecular Design of Redox-Active Carbonyl-Bridged Heterotriangulenes for High-Performance Lithium-Ion Batteries

Xipeng Shu,[1] Liang Hu,[1] Thomas Heine,[2,3,4,] and Yu Jing[1,*]*

X. Shu, L. Hu, Prof. Y. Jing
Jiangsu Co-Innovation Centre of Efficient Processing and Utilization of Forest Resources, College of Chemical Engineering, Nanjing Forestry University, Nanjing 210037, China
Email: yujing@njfu.edu.cn

Prof. T. Heine
TU Dresden, Fakultät für Chemie und Lebensmittelchemie, Bergstraße 66c, 01062 Dresden, Germany
Email: thomas.heine@tu-dresden.de

Prof. T. Heine
Helmholtz-Zentrum Dresden-Rossendorf, Forschungsstelle Leipzig, Permoserstraße 15, 04318 Leipzig, Germany

Prof. T. Heine
Department of Chemistry, Yonsei University, Seodaemun-gu, Seoul 120-749, Republic of Korea.

1. Computational details

The binding energy ($E_{binding}$) of lithium was calculated according to:

$$E_{binding}=(E_{M+nLi} - E_M - nE_{Li})/n, \quad (1)$$

where E_{M+nLi} and E_M are the total energies (corrected by zero-point energy) of CBHTs and their derivatives after and before binding with lithium, respectively (M represents CBHTs or their derivatives). E_{Li} is the total energy of a lithium atom from a Li-dimer.[1] The n denotes the number of combined lithium atoms in each molecule. The Gibbs free energies change (ΔG) of each lithiation step in the solution(electrolyte) can be calculated as:

$$\Delta G_{sol} = (G^{sol}_{M+nLi} - G^{sol}_{M} - nG^{sol}_{Li})/n, \quad (2)$$

where G^{sol}_{M+nLi}, G^{sol}_{M} and G^{sol}_{Li} represent the Gibbs free energies of CBHTs and their derivatives after and before binding with lithium, and lithium in solution, respectively.

Previous studies have demonstrated that DFT calculations can reliably predict the redox activity of carbonyl compounds[2,3] and give the redox potential of quinone molecules consistent with experiments. [4,5] In order to effectively examine the redox activity of different CBHTs, the redox potential ($E_{potential}$) of different molecules in the solution is calculated using the following equation:

$$E_{potential} = -\Delta G_{sol}/nF, \quad (3)$$

where F represents the Faraday constant (F = 96,485 C mol^{-1}) and n is the number of transferred electrons. The theoretical capacity (C_t) can be obtained by:

$$C_t = nF/3.6W_M, \quad (4)$$

where W_M represents the molecular weight of CBHTs or their derivatives. The molecular polarity index (MPI, kcal mol^{-1}) is defined by the following equation:

$$MPI = (1/A) \iint_S |V(r)|dS, \quad (5)$$

where A is the molecular surface area, V is the molecular electrostatic potential, and the integral is performed over the molecular surface. The higher the MPI value, the larger the overall polarity of the molecule. This index can be used to describe the polarity of molecules.

Note that to validate the reliability of the employed computational methods, we have calculated the redox potentials of fused N-heteroaromatic triquinoxalinylene molecules that exhibit similar skeletons to our studied molecules.[6] The calculated redox potential and theoretical capacity are in good agreement with the experimental results (Figure S1, Table S9), indicating that our prediction of the redox activity of CBHTs will be reliable.

The charge transport properties of CBHTs was described based on the hopping mechanism,[7] which supposes that each of these hops is considered as a non-adiabatic charge transfer reaction. Here, the hopping path is obtained by considering all possible stacking

patterns of CBHTs. By extracting the wave functions of all different dimers and generating the electron coupling terms of charge transfer through the dimer Hamiltonian model, the transfer integrals under all paths can be finally obtained. The hopping rate between two adjacent molecules was evaluated using the standard Marcus equation:[8]

$$k = \frac{V^2}{\hbar}\left(\frac{\pi}{\lambda k_B T}\right)^{1/2} \exp\left(-\frac{\lambda}{4k_B T}\right), \quad (6)$$

where λ is the reorganization energy, V is the intermolecular transfer integral, $\hbar$ is the Planck constant, and k_B is the Boltzmann constant. The reorganization energy associated with the charge transfer process is related to the local electron-phonon coupling, which is calculated by the adiabatic potential energy surface method (four-point method).[9] The intermolecular transfer integral was evaluated by the electronic coupling terms of the nearest neighbor molecules. The electronic coupling for charge transfer can be obtained by using the dimer Hamiltonian model:[10]

$$V=\langle \phi_{HOMO}^{0,\ site\ 1} | F | \phi_{HOMO}^{0,\ site\ 2} \rangle, \quad (7)$$

where $\phi_{HOMO}^{0,\ site\ 1}$ and $\phi_{HOMO}^{0,\ site\ 2}$ indicate the HOMOs of isolated molecules 1 and 2, respectively, and F is the Fock operator for the dimer. Charge transport can be described by the Brownian motion process. The mobility can be expressed by the Einstein equation:[11]

$$\mu = \frac{e}{k_B T} D, \quad (8)$$

where D represents the isotropic charge diffusion constant, which can be calculated as:[12]

$$D = \frac{1}{2n} \sum_i r_i^2 k_i P_i, \quad (9)$$

Here, n is the dimension, r_i is the distance from the central molecule to the i_{th} neighboring molecule, k_i is the hopping rate of the i_{th} hopping path, and P_i is the probability of the charge following the i_{th} hopping path:

$$P_i = k_i / \sum_i k_i, \quad (10)$$

2. Electrochemical performance of CBHTs and their N-functionalized derivatives

To verify the reliability of the computational method used in this work, we used the redox potentials of fused N-heteroaromatic triquinoxalinylene molecules (3Q) that exhibits a similar skeleton to our molecules as a benchmark. The average redox potential of 3Q is calculated to be 1.93 V (*vs* Li^+/Li), which is only 0.17 V higher than the cyclic voltammetry measured average reduction potential (1.76 V, *vs* Li^+/Li),[6] demonstrating the reliability of our calculations.

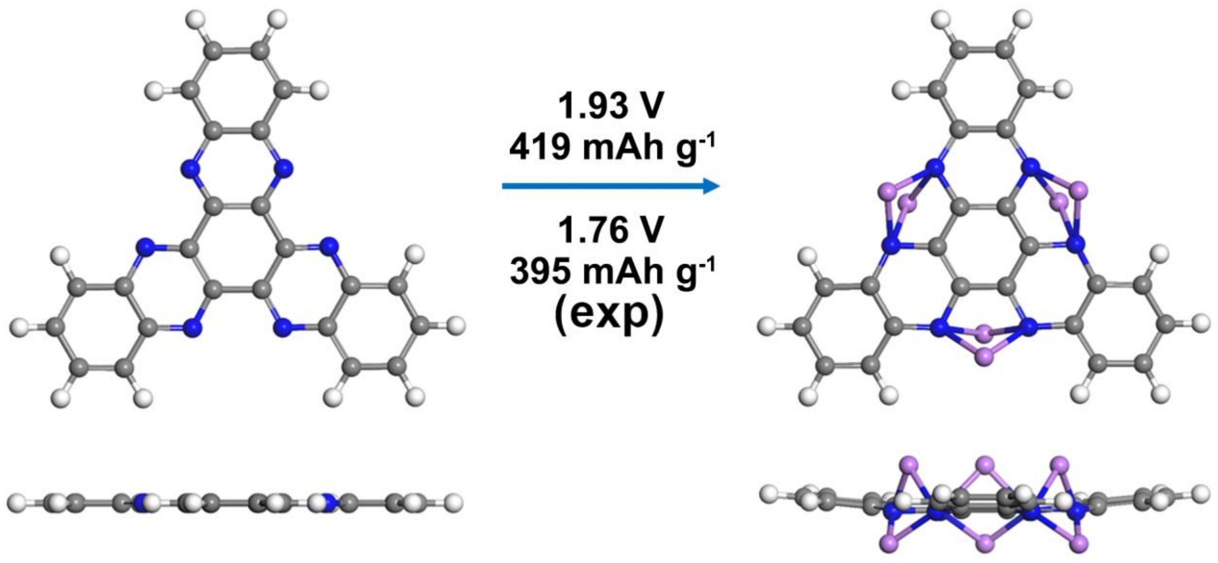

Figure S1. Optimized molecular structure of 3Q before and after combining with the maximum amount of Li. The gray, white, blue, and violet balls represent C, H, N, and Li, respectively

Table S1. Energy level of the highest occupied molecular orbital (HOMO) and the lowest unoccupied molecular orbital (LUMO) and HOMO-LUMO gap (E_{gap}) of CBHTs (all values are in eV).

Structures	HOMO	LUMO	E_{gap}
CTPB	-7.05	-3.42	3.63
CTPA	-6.38	-2.58	3.80
CTPP	-6.69	-2.50	4.19

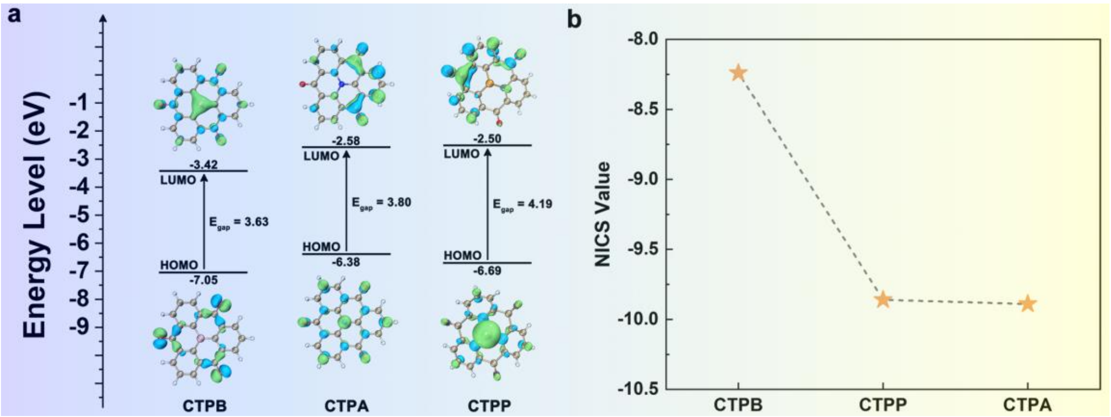

Figure S2. (a) The energy level (eV), shape of frontier orbitals and (b) nucleus-independent chemical shift (NICS) for CTPB, CTPA and CTPP, respectively.

The p_z orbital of the central N/P atoms in CTPA/P is occupied with a pair of electrons, while the B of CTPB shows an empty p_z orbital. As a result, the B center of CTPB and the N/P

center of CTPA/P show singular and opposite contribution to the LUMO and HOMO orbitals, respectively, leading to different shape of the frontier orbitals. Moreover, as revealed by NICS results (Figure S2 b), the empty p_Z orbital of central B also contributed to reduced aromaticity of CTPB compared with that of CTPA/P.

Table S2. The 1st Li-binding energy ($E_{bingding}$, eV), theoretical specific capacity (Ct, unit: mAh g-1), average redox potential ($E_{ave,sol}$, unit: V) and energy density (E_d, Wh kg^{-1}) for original CBHTs.

Structures	$E_{binding}$	$E_{ave,sol}$	C_t	E_d
CTPB	-1.78	2.08	251.26	522.81
CTPA	-1.43	1.83	248.93	454.41
CTPP	-1.40	1.79	236.48	423.30

Table S3. The energy level of HOMO and LUMO (eV), HOMO-LUMO gap (E_{gap}, eV), Li-binding energy ($E_{bingding}$, eV) and redox potential ($E_{nLi,sol}$, unit: V) for CTPB after combining with nLi (n = 1, 2 or 3).

Structures	HOMO	LUMO	E_{gap}	$E_{binding}$	$E_{nLi,sol}$
CTPB-1Li	-4.06	-2.21	1.85	-1.78	2.48
CTPB-2Li	-3.37	-2.09	1.28	-1.43	2.18
CTPB-3Li	-2.55	-1.51	1.04	-1.03	1.59

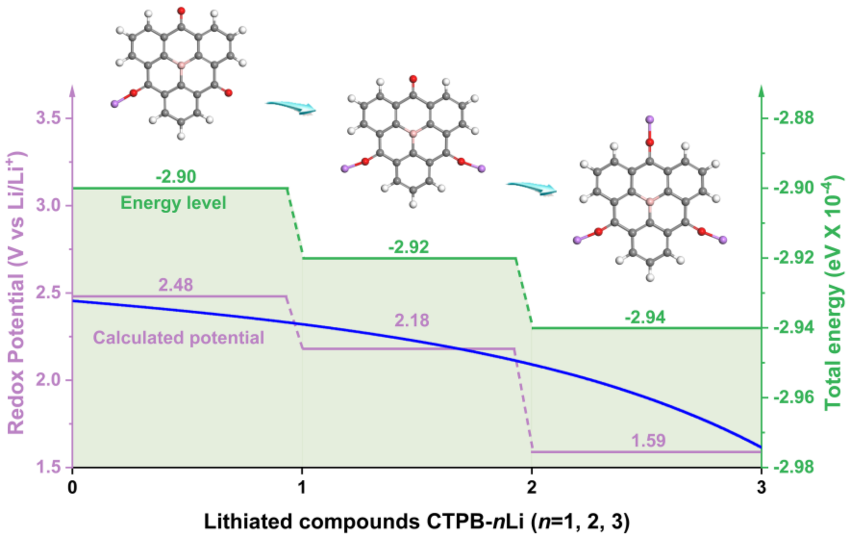

Figure S3. The optimized lithiated sequence and structures of CTPA after lithiation. The changes of calculated potentials (purple line), simulated discharge curve (blue line) and binding energy (green line) during the lithiation processes of CTPA.

Table S4. Previously reported electrochemical performance of carbonyl compounds as cathode materials for LIBs.

Cathode	Redox potential (V)	Specific capacity (mAh g^{-1})	Ref.
DMBQ	2.60	319	[13]
PADQ	2.63	403	[14]
DANQ	2.30	250	[15]
BFFD	2.45	234	[16]

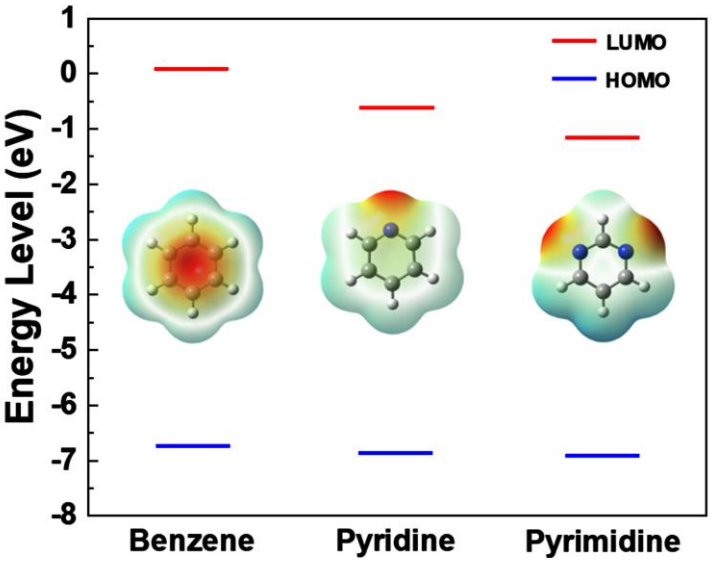

Figure S4. Energy level (eV) of frontier orbitals and MESP distribution for benzene, pyridine and pyrimidine, respectively. The red and blue colors indicate regions with negative and positive electronic potential, respectively.

The result is consistent with a previous study,[17,18] the LUMO and HOMO energy levels shift downward with increasing the number of pyridinic N, and the HOMO-LUMO gap decreases.

Table S5. Energy level of HOMO and LUMO and HOMO-LUMO gap (E_{gap}) of N-functionalized CBHTs. Values are in eV.

Structures	HOMO	LUMO	E_{gap}
CTPB(4-pyridyl)	-7.28	-4.29	2.99
CTPB(3-pyridyl)	-7.30	-3.89	3.41
CTPB(3,5-pyrimidyl)	-7.52	-4.42	3.09
CTPA(4-pyridyl)	-7.44	-3.21	4.23

CTPA(3-pyridyl)	-6.89	-3.27	3.62
CTPA(3,5-pyrimidyl)	-7.48	-4.00	3.48
CTPP(4-pyridyl)	-7.37	-3.09	4.28
CTPP(3-pyridyl)	-7.10	-3.13	3.98
CTPP(3,5-pyrimidyl)	-7.92	-4.21	3.71

Table S6. The 1st Li-binding energy ($E_{bingding}$, eV), theoretical specific capacity (Ct, unit: mAh g-1), average redox potential ($E_{ave,sol}$, V) and energy density (E_d, Wh kg^{-1}) of different N-functionalized CBHTs.

Structures	$E_{binding}$	$E_{ave,sol}$	C_t	E_d
CTPB(4-pyridyl)	-2.20	2.38	248.93	593.11
CTPB(3-pyridyl)	-3.08	3.01	248.93	749.28
CTPB(3,5-pyrimidyl)	-3.30	3.09	493.28	1524.24
CTPA(4-pyridyl)	-1.70	1.99	246.64	491.79
CTPA(3-pyridyl)	-2.94	2.94	246.64	725.12
CTPA(3,5-pyrimidyl)	-3.27	2.56	488.78	1251.28
CTPP(4-pyridyl)	-1.66	1.84	234.42	431.33
CTPP(3-pyridyl)	-2.82	2.80	234.42	656.38
CTPP(3,5-pyrimidyl)	-3.09	2.81	467.77	1306.01

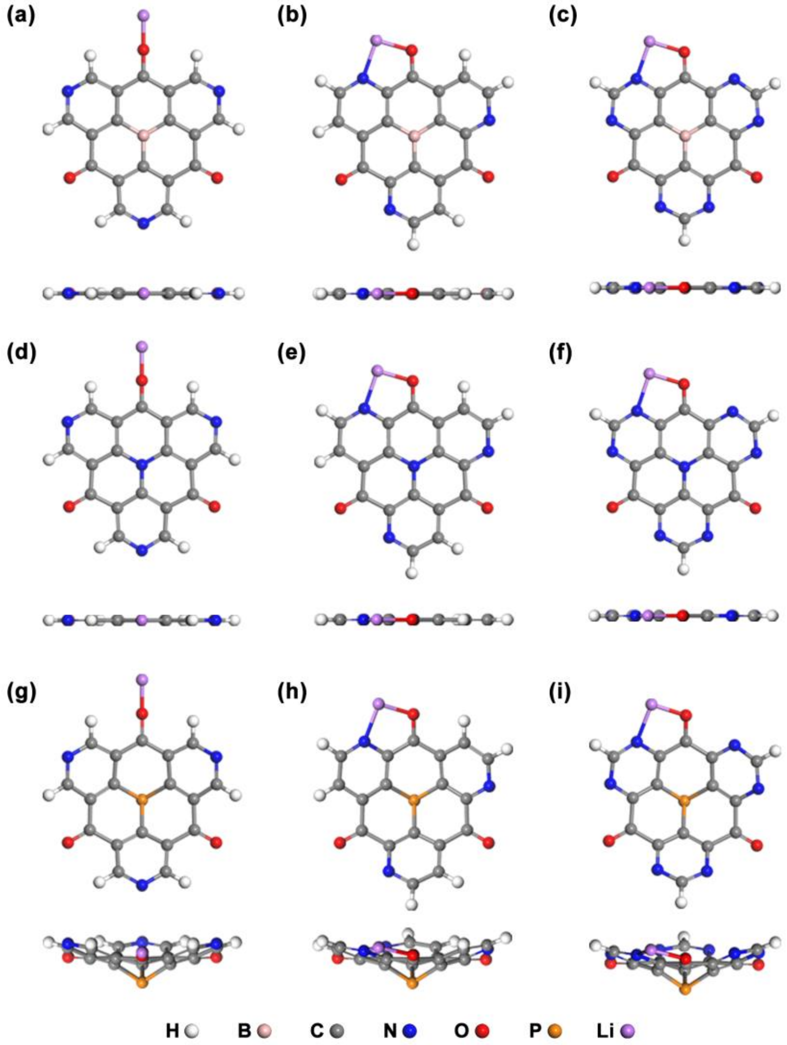

Figure S5. Optimized molecular structures combined with one Li (1st-Li) at the optimal site for (a) carbonyl-bridged tri (4-pyridyl) borane (CTPB(4-pyridyl)), (b) carbonyl-bridged tri (3-pyridyl) borane (CTPB(3-pyridyl)), (c) carbonyl-bridged tri (3,5-pyrimidyl) borane (CTPB(3,5-pyrimidyl)), (d) carbonyl-bridged tri (4-pyridyl) amine (CTPA(4-pyridyl)), (e) carbonyl-bridged tri (3-pyridyl) amine (CTPA(3-pyridyl)), (f) carbonyl-bridged tri (3,5-pyrimidyl) amine (CTPA(3,5-pyrimidyl)), (g) carbonyl-bridged tri (4-pyridyl) phosphine (CTPP(4-pyridyl)), (h) carbonyl-bridged tri (3-pyridyl) phosphine (CTPP(3-pyridyl)), and (i) carbonyl-bridged tri (3,5-pyrimidyl) phosphine (CTPP(3,5-pyrimidyl)), respectively.

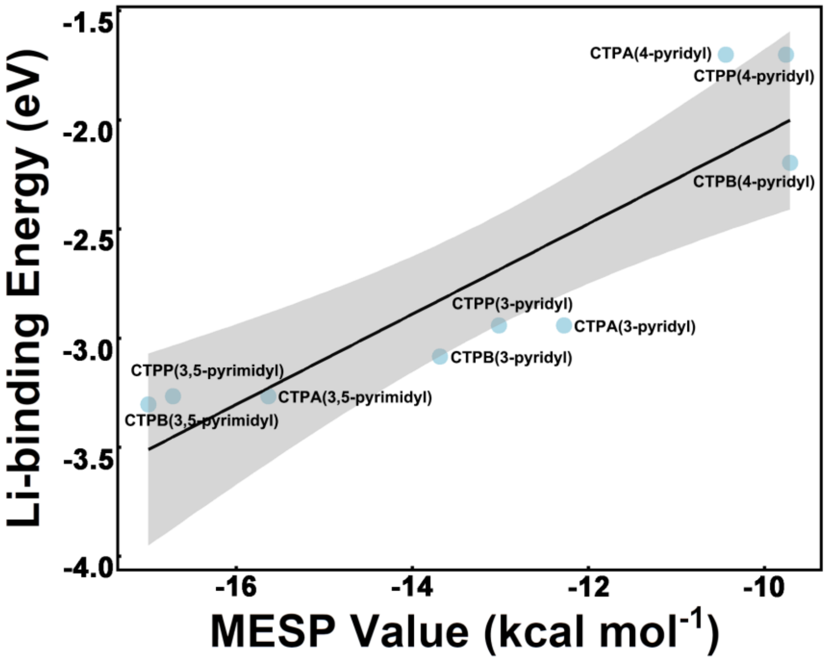

Figure S6. Correlations of the 1st Li-binding energy with the MESP value for different N-functionalized CBHTs ($R = 0.89$, $P = 0.0012$). Note that R is the Pearson correlation coefficient, which measures the degree of linear correlation between the two variables. P is obtained using the significance test method to determine the correlation degree. Usually, a P value of less than 0.05 indicates a significant correlation between the two variables.

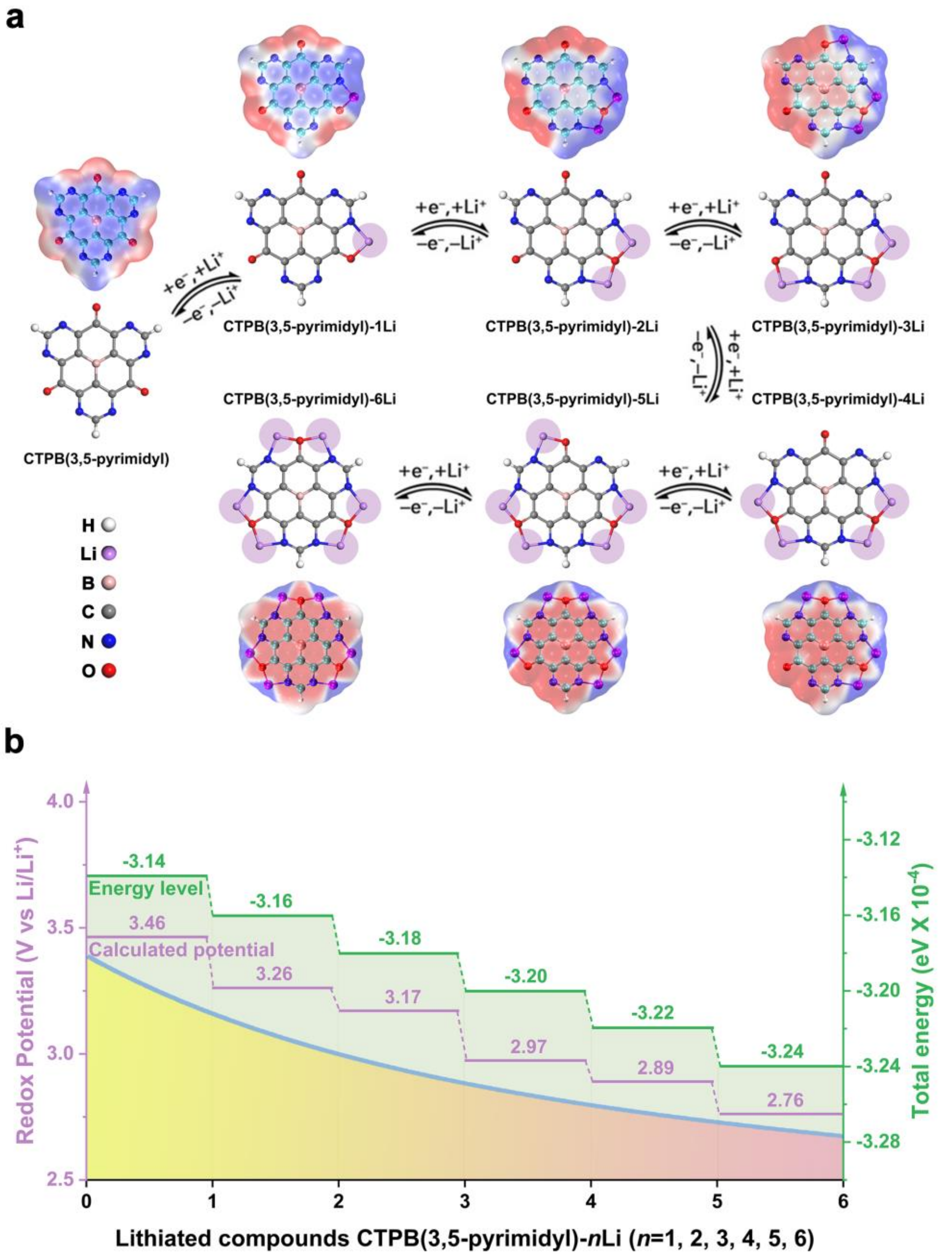

Figure S7. (a) The optimized lithiated sequence and structures of CTPB(3,5-pyrimidyl) after lithiation, (b) The changes of calculated potentials (purple line), simulated potential curve (blue line) and total energy (green line) during the lithiation processes of CTPB(3,5-pyrimidyl).

As illustrated in Table S7 and Figure S7, the bridge sites between the carbonyl O and pyridinic N are always the most favorable sites for Li binding, which is confirmed by examining the binding energy. Moreover, the discharge curves obtained from a step-wise and a simultaneous process show the identical decreasing trend and the calculated average redox potential in these two ways is also the same.

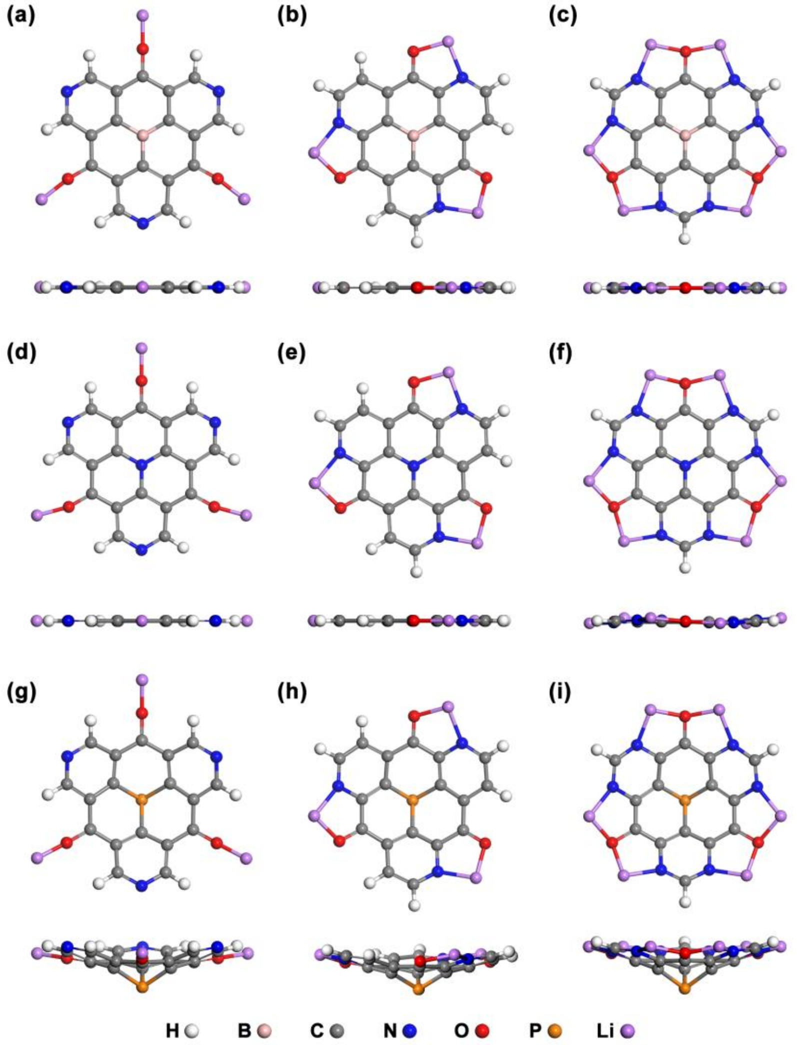

Figure S8. Optimized molecular structures combined with the maximum amount of Li (Max-Li) for (a) CTPB(4-pyridyl)), (b)CTPB(3-pyridyl), (c)CTPB(3,5-pyrimidyl), (d) CTPA(4-pyridyl), (e) CTPA(3-pyridyl), (f)CTPA(3,5-pyrimidyl), (g)CTPP(4-pyridyl), (h)CTPP(3-pyridyl) and (i)CTPP(3,5-pyrimidyl), respectively.

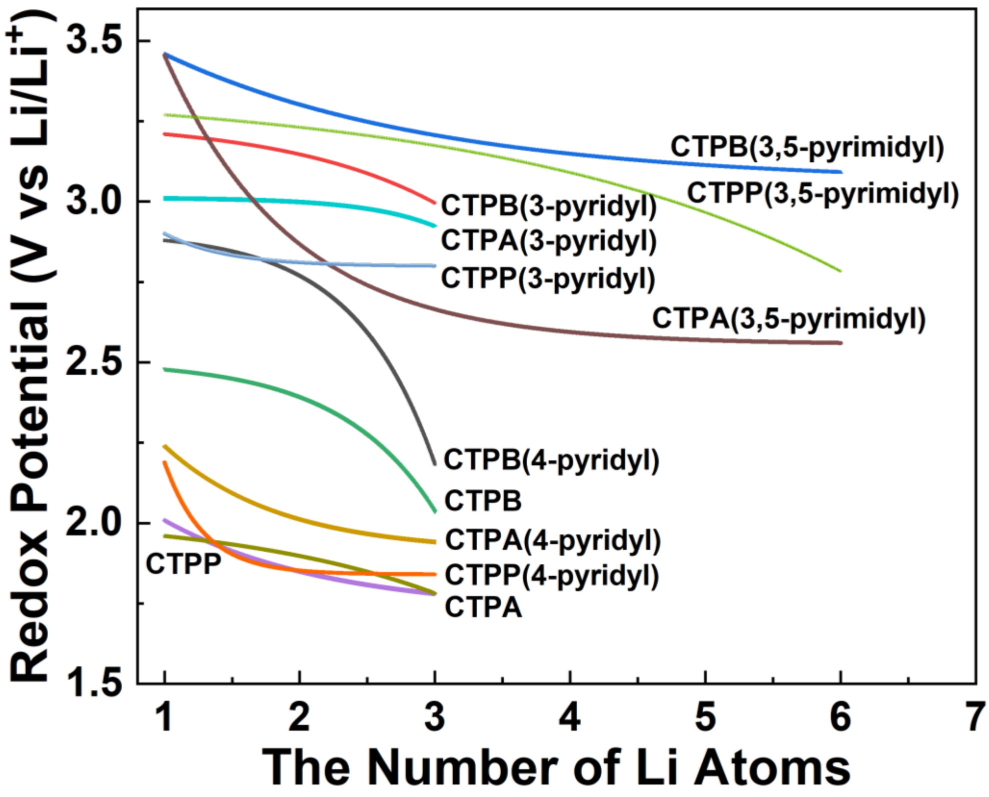

Figure S9. The change of the redox potential of CBHTs and their N-functionalized derivatives with the increase of the amount of combined Li.

Table S7. The energy level of HOMO and LUMO (eV), HOMO-LUMO gap (E_{gap}, eV), Li-binding energy ($E_{bingding}$, eV) and redox potential ($E_{nLi,sol}$, unit: V) of CTPB(3,5-pyrimidyl) after combining with nLi (n= 1, 2, 3, 4, 5 or 6).

Structures	HOMO	LUMO	E_{gap}	$E_{binding}$	$E_{nLi,sol}$
CTPB(3,5-pyrimidyl)-1Li	-5.68	-4.02	1.66	-3.27	3.46
CTPB(3,5-pyrimidyl)-2Li	-5.23	-3.41	1.81	-3.27	3.26
CTPB(3,5-pyrimidyl)-3Li	-4.55	-2.72	1.83	-2.99	3.17
CTPB(3,5-pyrimidyl)-4Li	-4.14	-2.30	1.84	-2.99	2.97
CTPB(3,5-pyrimidyl)-5Li	-3.56	-1.89	1.67	-2.72	2.89
CTPB(3,5-pyrimidyl)-6Li	-2.84	-0.48	2.36	-2.72	2.76

Table S8. The mass energy density of CTPB/A/P(3,5-pyrimidyl) in comparison with that of other organic cathode materials.

Structures	Energy density (Wh kg^{-1})	Ref.
CTPB(3,5-pyrimidyl)	1524.24	This work
CTPA(3,5-pyrimidyl)	1251.28	This work
CTPP(3,5-pyrimidyl)	1524.24	This work
3Q	780.12	maintext ref. 24
PDDTB	831.60	maintext ref. 55
PBQS	734.25	maintext ref. 54
DCA	330.00	maintext ref. 56
ADALS	275.50	maintext ref. 57

Table S9. The theoretically calculated capacity of previously reported organic cathode materials in comparison with the experimentally measured values.

Cathode	Capacity (exp, mAh g^{-1})	Capacity (theo, mAh g^{-1})	Ref.
3Q	394	419	maintext ref. 24
PDDTB	378	375	maintext ref. 55
PBQS	275	311	maintext ref. 54
DCA	200	235	maintext ref. 56
ADALS	190	193	maintext ref. 57

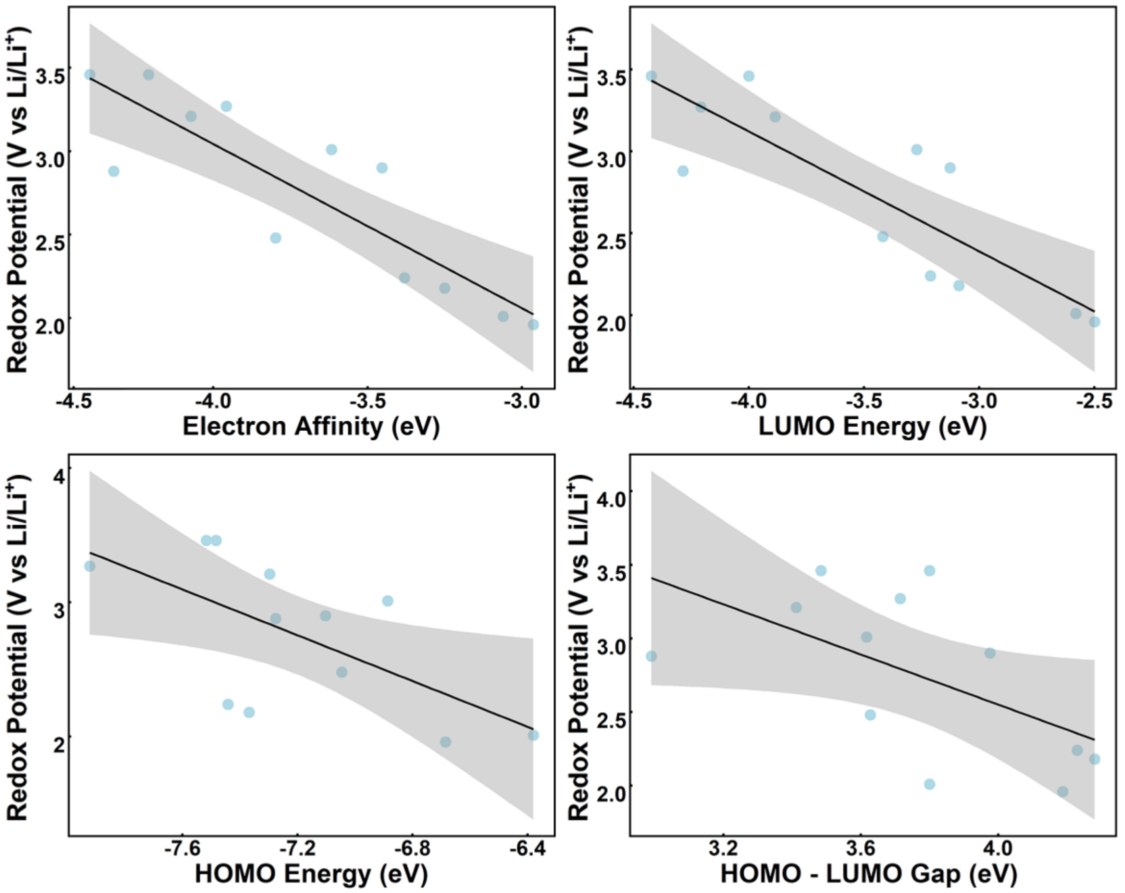

Figure S10. Correlations of the calculated redox potential with the (a) electron affinity (R = -0.87, P = 0.00023), (b) LUMO energy level (R = -0.85, P = 0.0004), (c) HOMO energy level (R = -0.63, P = 0.028), and (d) LUMO-HOMO gap (R = -0.57, P = 0.051) for CBHTs and their N-functionalized derivatives.

To better understand the underlying structure-activity relationship of different CBHTs and their derivatives, we investigated the correlation between the electronic properties including electron affinity, HOMO, LUMO, HOMO–LUMO gap and the redox potential. As shown in **Figure S10a**, there is an obvious linear relationship between the redox potential and electron affinity indicating that the higher redox potential of N-functionalized CBHTs stems from their stronger electron affinity. This result is consistent with a previous study,[19] demonstrating that molecules with higher electron affinity obtain electrons more readily and consequently show higher redox potentials. **Figure S10b** shows a linear relationship between the LUMO levels and the redox potentials, so molecules with deeper LUMO position show higher redox potential, demonstrating that the lower the LUMO energy level of the organic molecules, the stronger the ability to obtain electrons and the higher the redox potentials.[20,21] Thus, CTPB(3,5-pyrimidyl) with the lowest LUMO energy level shows the highest redox potential. For other electronic properties, such as the HOMO levels and the HOMO–LUMO gaps (**Figure S10c,d**), no significant correlation with the redox potentials was observed.

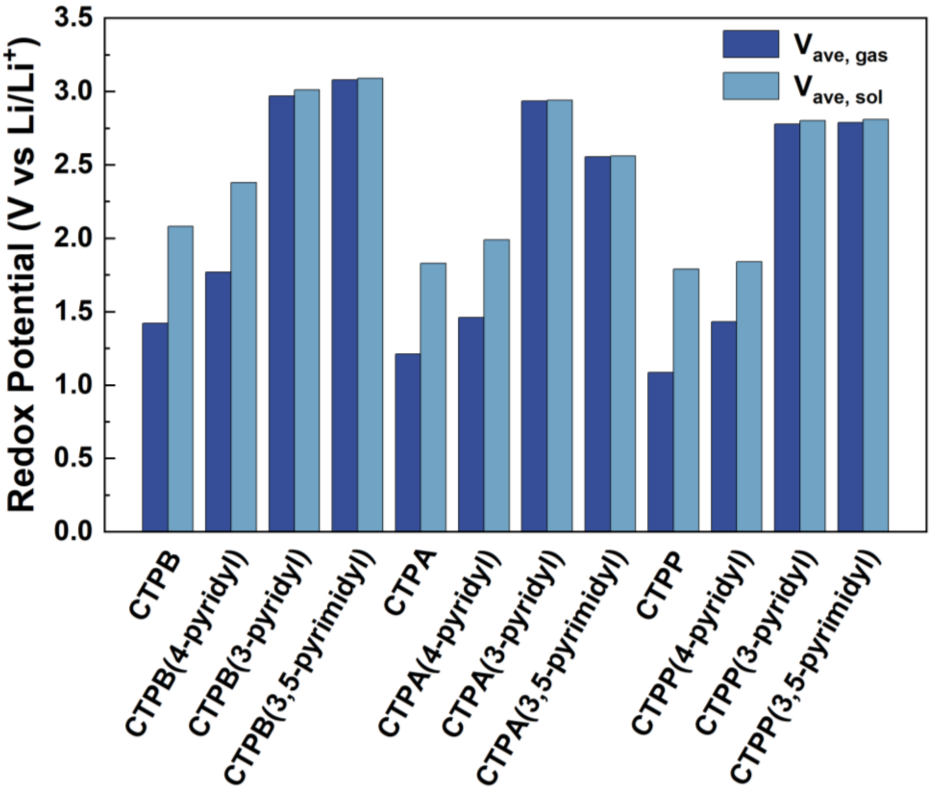

Figure S11. The calculated redox potential of CBHTs and their N-functionalized derivatives in vacuum and solution of electrolyte (EC: DMC, 3:7 by volume).

Table S10. The calculated average redox potentials (V) in vacuum ($E_{ave,gas}$) and three solution conditions.[a)]

Structures	$E_{ave,gas}$	$E_{ave,sol1}$	$E_{ave,sol2}$	$E_{ave,sol3}$
CTPB	1.42	2.00	2.08	2.08
CTPB(4-pyridyl)	1.77	2.28	2.38	2.38
CTPB(3-pyridyl)	2.97	3.01	3.01	3.01
CTPB(3,5-pyrimidyl)	3.08	3.09	3.09	3.09
CTPA	1.21	1.79	1.83	1.83
CTPA(4-pyridyl)	1.46	1.94	1.99	1.99
CTPA(3-pyridyl)	2.93	2.94	2.94	2.94
CTPA(3,5-pyrimidyl)	2.55	2.56	2.56	2.56
CTPP	1.08	1.70	1.79	1.79
CTPP(4-pyridyl)	1.43	1.79	1.84	1.84
CTPP(3-pyridyl)	2.78	2.79	2.80	2.80
CTPP(3,5-pyrimidyl)	2.79	2.79	2.81	2.81

[a)]The most common ethylene carbonate (EC) and dimethyl carbonate (DMC) (3:7 v/v), 1,2-dimethoxyethane (DME) and 1,1,3,3-tetramethylurea (TMU) were used as the electrolyte to estimate the solvation effect. The solvent 1, solvent 2 and solvent 3 represent DME (ε_r =7.2), EC and DMC (3:7 v/v, ε_r = 16.14) and TMU (ε_r = 24.46), respectively.

Table S11: Polar surface area and molecular polarity index (MPI, kcal mol^{-1}) of CBHTs and their N-functionalized derivatives in comparison with those of 3Q.

Structures	Polar surface area	MPI
CTPB	39.61%	8.81
CTPB(4-pyridyl)	68.22%	12.89
CTPB(3-pyridyl)	70.01%	15.07
CTPB(3,5-pyrimidyl)	79.19%	18.38
CTPA	45.27%	10.16
CTPA(4-pyridyl)	72.37%	14.14
CTPA(3-pyridyl)	75.44%	16.32
CTPA(3,5-pyrimidyl)	80.01%	19.55
CTPP	39.75%	9.31
CTPP(4-pyridyl)	66.69%	13.15
CTPP(3-pyridyl)	70.05%	14.87
CTPP(3,5-pyrimidyl)	80.14%	18.81
3Q	34.58%	9.64

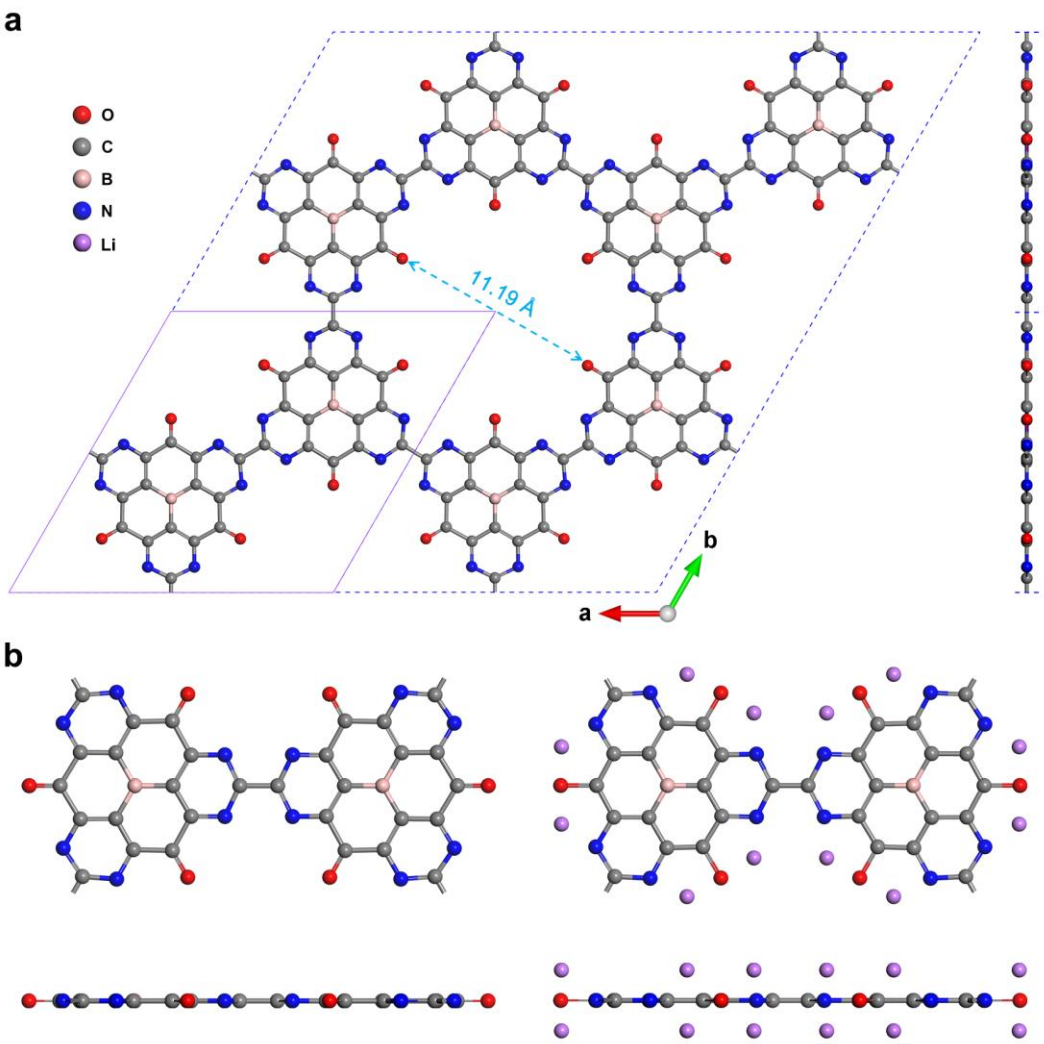

Figure S12. (a) Top and side views of a structurally optimized 2D CTPB(3,5-pyrimidyl) in a 2×2×1 supercell, the optimized lattice parameter of 2D CTPB(3,5-pyrimidyl) is a = b = 16.90 Å, and the pore size is 11.19 Å. (b) Optimized molecular structure of 2D- CTPB(3,5-pyrimidyl) before and after combining with the maximum amount of Li.

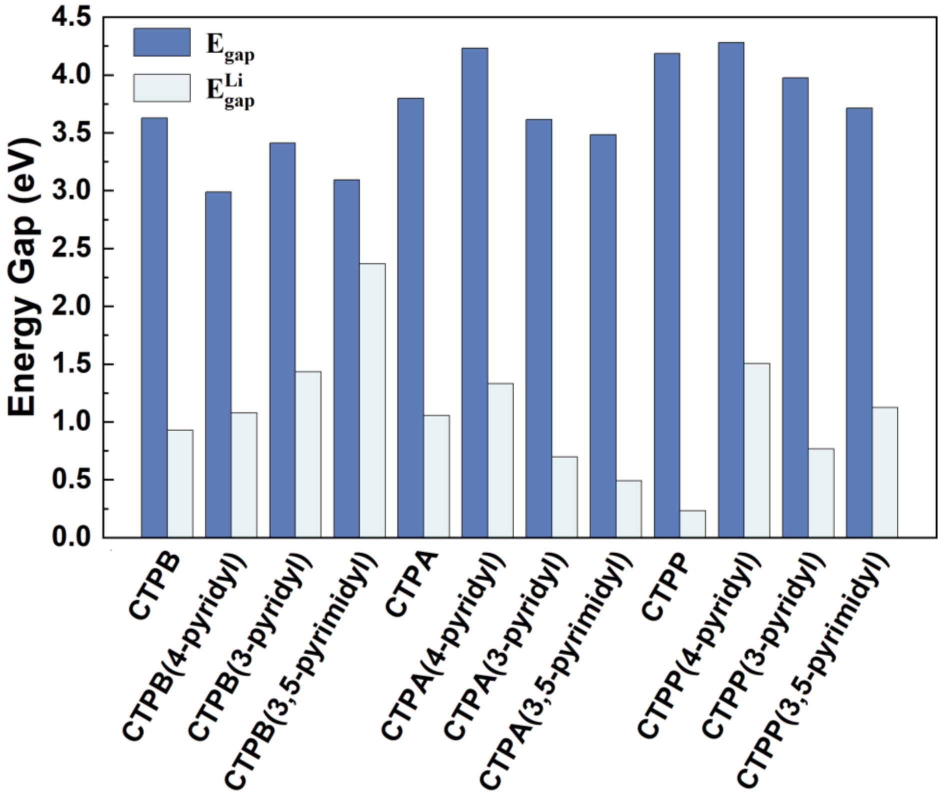

Figure S13. The HOMO–LUMO gap for CBHTs and their N-functionalized derivatives before and after lithiation.

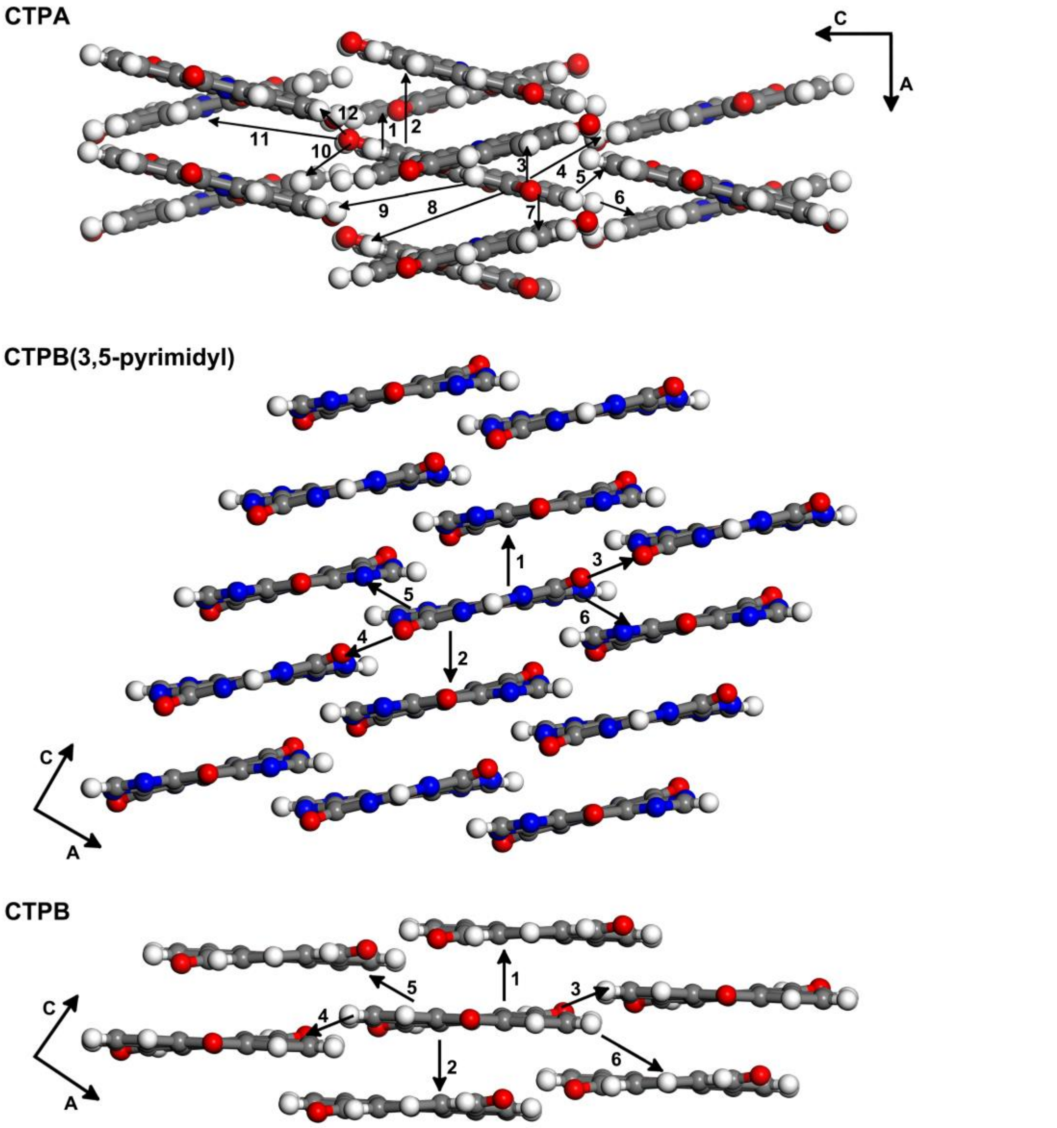

Figure S14. Schemes of charge hopping pathways for CTPA, CTPB(3,5-pyrimidyl) and CTPB.

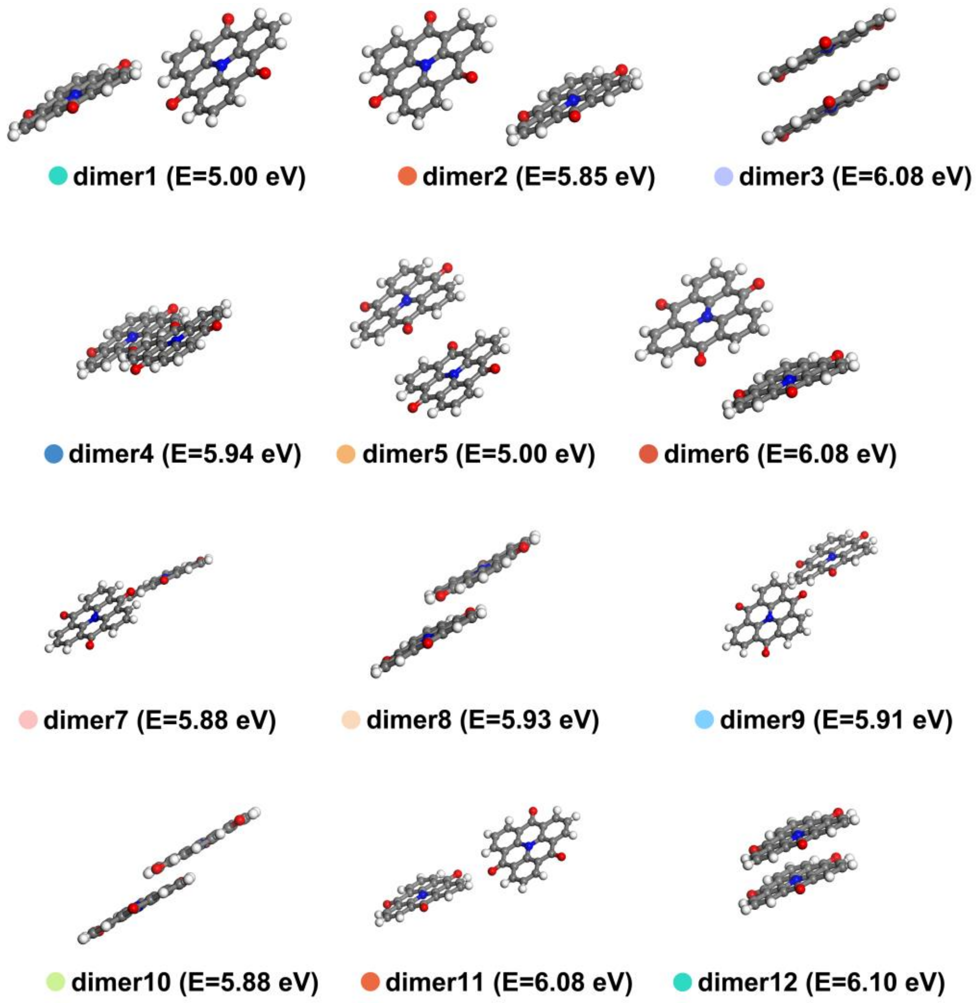

Figure S15. Prototypical dimer configurations for CTPA. The formation energy ($E= E_{dimer}-2E_{monomer}$, eV) of CTPA dimer configurations are given in parentheses.

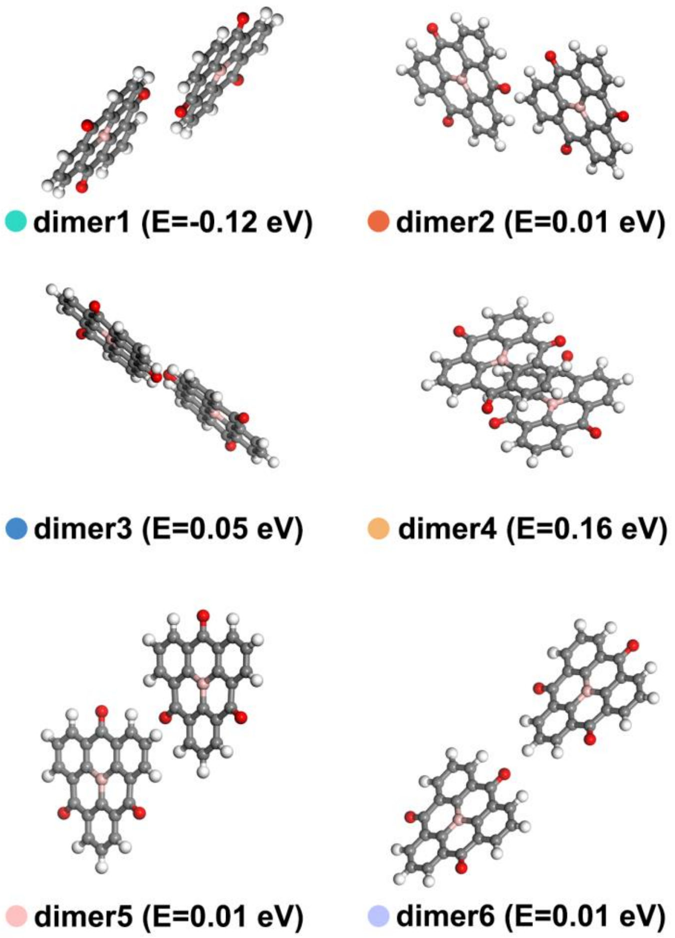

Figure S16. Prototypical dimer configurations for CTPB with the formation energy (eV) given in the parentheses.

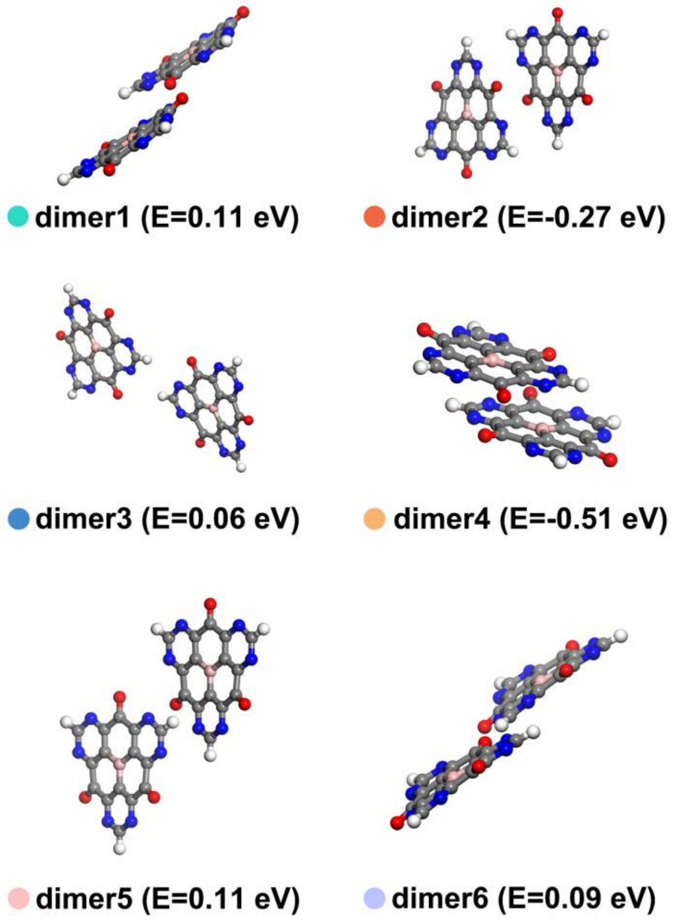

Figure S17. Prototypical dimer configurations of CTPB(3,5-pyrimidyl). The formation energy (eV) of different CTPB(3,5-pyrimidyl) dimers is given in the parentheses.

Table S12. Change in bond length (pm) of the central (C-B, C-N or C-P) bond and carbonyl (C=O) bridging bond of CBHTs and their N-functionalized derivatives after lithiation.

Structures	central bond (pm)	carbonyl bond (pm)
CTPB	4.19	11.03
CTPB(4-pyridyl)	3.74	10.51
CTPB(3-pyridyl)	2.02	7.95
CTPB(3,5-pyrimidyl)	3.78	18.10
CTPA	0.29	6.36
CTPA(4-pyridyl)	0.27	10.40
CTPA(3-pyridyl)	0.62	8.20
CTPA(3,5-pyrimidyl)	0.46	13.42
CTPP	2.67	6.43
CTPP(4-pyridyl)	3.86	11.39
CTPP(3-pyridyl)	2.34	7.99
CTPP(3,5-pyrimidyl)	1.03	18.80

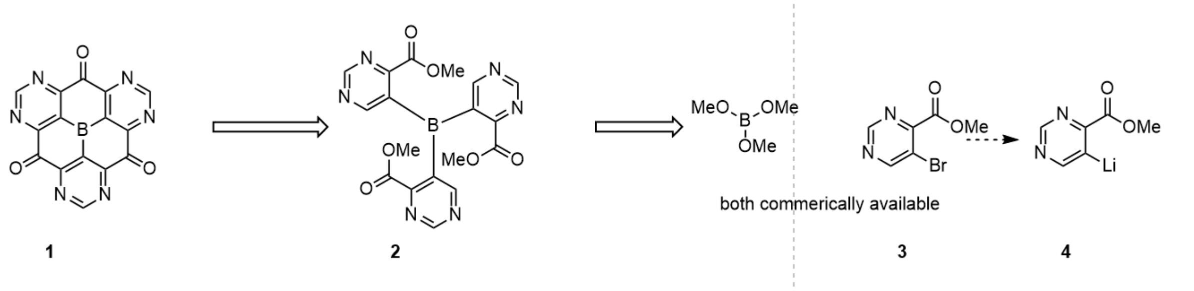

Scheme S1. The possible synthetic route for the target CTPB(3,5-pyrimidyl) molecule.

From retrosynthetic perspective, the target compound 1 could be constructed from tri-ester 2 via Friedel-Crafts reaction in one step. The tri-ester 2 could be easily obtained via nucleophilic addition toward trimethyl borate with in situ generated lithium reagent 4, which could be prepared from bromide 3 through lithium-halogen exchange. Both trimethyl borate and bromide 3 are commercially available.

References

[1] Z. Li, Q. Jia, Y. Chen, K. Fan, C. Zhang, G. Zhang, M. Xu, M. Mao, J. Ma, W. Hu, C. Wang, *Angew. Chem. Int. Ed.* **2022,** *61*, e202207221.

[2] S. Er, C. Suh, M. P. Marshak, A. Aspuru-Guzik, *Chem. Sci.* **2015**, *6*, 885.

[3] J. E. Bachman, L. A. Curtiss, R. S. Assary, *J. Phys. Chem. A* **2014**, *118*, 8852.

[4] K. C. Kim, T. Liu, S. W. Lee, S. S. Jang, *J. Am. Chem. Soc.* **2016**, *138*, 2374.

[5] X. Zhu, Y. Jing, *J. Power Sources* **2022**, *531*, 231291.

[6] C. Peng, G.-H. Ning, J. Su, G. Zhong, W. Tang, B. Tian, C. Su, D. Yu, L. Zu, J. Yang, M.-F. Ng, Y.-S. Hu, Y. Yang, M. Armand, K. P. Loh, *Nat. Energy* **2017**, *2*, 17074.

[7] V. Coropceanu, J. Cornil, D. A. da Silva Filho, Y. Olivier, R. Silbey, J.-L. Brédas, *Chem. Rev.* **2007**, *107*, 926.

[8] R. A. Marcus, *Angew. Chem. Int. Ed.* **1993**, *32*, 1111.

[9] M. Malagoli, J. L. Brédas, *Chem. Phys. Lett.* **2000**, *327*, 13.

[10] A. Troisi, G. Orlandi, *Chem. Phys. Lett.* **2001**, *344*, 509.

[11] L. B. Schein, A. R. McGhie, *Phys. Rev. B.* **1979**, *20*, 1631.

[12] S. Chai, S. H. Wen, J. D. Huang, K. L. Han, *J. Comput. Chem.* **2011**, *32*, 3218.

[13] M. Yao, H. Senoh, S.-i. Yamazaki, Z. Siroma, T. Sakai, K. Yasuda, *J. Power Sources* **2010**, *195*, 8336.

[14] D. Wu, Z. Xie, Z. Zhou, P. Shen, Z. Chen, *J. Mater. Chem. A* **2015**, *3*, 19137.

[15] J. Lee, H. Kim, M. J. Park, *Chem. Mater.* **2016**, *28*, 2408.

[16] Y. Liang, P. Zhang, S. Yang, Z. Tao, J. Chen, *Adv. Energy Mater.* **2013**, *3*, 600.

[17] D. Chen, S.-J. Su, Y. Cao, *J. Mater. Chem. C* **2014,** *2*, 9565.

[18] S. Krotkus, T. Matulaitis, S. Diesing, Zysman-Colman, I. D. W. Samuel, *Front. Chem.* **2020,** *8*, 572862.

[19] J. H. Park, T. Liu, K. C. Kim, S. W. Lee, S. S. Jang, *ChemSusChem* **2017**, *10*, 1584.

[20] Y. Liang, P. Zhang, S. Yang, Z. Tao, J. Chen, *Adv. Energy Mater.* **2013**, *3*, 600.

[21] Chang-Guo. Zhan, Jeffrey. A. Nichols, David. A. Dixon, J. Chen, *J. Phys. Chem. A* **2003**, *107*, 4184.
